# Prevalence of Headache in Patients With Coronavirus Disease 2019 (COVID-19): A Systematic Review and Meta-Analysis of 14,275 Patients

**DOI:** 10.3389/fneur.2020.562634

**Published:** 2020-11-27

**Authors:** Md Asiful Islam, Sayeda Sadia Alam, Shoumik Kundu, Tareq Hossan, Mohammad Amjad Kamal, Cinzia Cavestro

**Affiliations:** ^1^Department of Hematology, School of Medical Sciences, Universiti Sains Malaysia, Kubang Kerian, Malaysia; ^2^Department of Biochemistry and Molecular Biology, Jahangirnagar University, Savar, Bangladesh; ^3^Department of Biochemistry and Molecular Biology, Cumming School of Medicine, University of Calgary, Calgary, AB, Canada; ^4^West China School of Nursing, Frontiers Science Center for Disease-Related Molecular Network, West China Hospital, Institutes for Systems Genetics, Sichuan University, Chengdu, China; ^5^King Fahd Medical Research Center, King Abdulaziz University, Jeddah, Saudi Arabia; ^6^Enzymoics, Novel Global Community Educational Foundation, Hebersham, NSW, Australia; ^7^Department of Neurology, Headache Centre, San Lazzaro Hospital, Alba, Italy

**Keywords:** coronavirus, COVID-19, headache, clinical, systematic review, meta-analysis

## Abstract

**Background:** Coronavirus disease 2019 (COVID-19) started to spread globally since December 2019 from Wuhan, China. Headache has been observed as one of the clinical manifestations in COVID-19 patients. We aimed to conduct a comprehensive systematic review and meta-analysis to estimate the overall pooled prevalence of headache in COVID-19 patients.

**Methods:** PubMed, Scopus, ScienceDirect, and Google Scholar databases were searched to identify studies published between December 2019 and March 2020. Adult (≥18 years) COVID-19 patients were considered eligible. We used random-effects model to estimate the pooled prevalence with 95% confidence intervals (CIs). Quality assessment was done using the Joanna Briggs Institute critical appraisal tools. This study is registered with PROSPERO (CRD42020182529).

**Results:** We identified 2,055 studies, of which 86 studies (*n* = 14,275, 49.4% female) were included in the meta-analysis. Overall, the pooled prevalence of headache in COVID-19 patients was 10.1% [95% CI: 8.76–11.49]. There was no significant difference of headache prevalence in severe or critical vs. non-severe (RR: 1.05, *p* = 0.78), survived (recovered or discharged) vs. non-survived (RR: 1.36, *p* = 0.23), and ICU vs. non-ICU (RR: 1.06, *p* = 0.87) COVID-19 patients. We detected 64.0, 34.9, and 1.1% of the included studies as high, moderate, and low quality, respectively.

**Conclusions:** From the first 4-month data of the outbreak, headache was detected in 10.1% of the adult COVID-19 patients.

## Introduction

In December 2019, a novel coronavirus, namely, severe acute respiratory syndrome coronavirus-2 (SARS-CoV-2), infection broke out in Wuhan, Hubei province, China, causing coronavirus disease 2019 (COVID-19) (1). Although it started in China, within a very short period of time, this infection has spread all over the world. Over 35 million people across 235 countries were infected with above 1 million confirmed death cases until 6th October, 2020 (2).

In the last 17 years, two other human coronaviruses, namely, SARS-CoV in November 2002 and Middle East respiratory syndrome coronavirus (MERS-CoV) in April 2012, were reported to cause SARS and MERS diseases, respectively, leading to a fatal lower respiratory tract infection (3, 4). Even though both SARS-CoV and MERS-CoV are closely linked to SARS-CoV-2, the evidence suggests that SARS-CoV-2 is more infectious and spreads more rapidly than that of SARS-CoV and MERS-CoV (5). A widespread clinical spectrum of SARS-CoV-2 infection has been observed ranging from asymptomatic, mild upper respiratory tract illness, to severe viral pneumonia with respiratory failure and death (6, 7). The clinical symptoms of COVID-19 include fever, cough, sore throat, muscle ache, shortness of breath, and headache (7–11).

Headache is a frequently observed symptom in several infectious diseases expressing intracranial inflammatory reaction (12). It can appear as the first symptom in meningeal involvement in these cases. Presence of headache was linked to other central nervous system (CNS) manifestations in patients with COVID-19, suggesting that it is also a CNS-associated infectious disease (13). Headache in patients with COVID-19 does not seem to have any particular characteristics, as it is described as tension-type headache or migraine without aura (14)—the two most frequently observed types of headache (12), or a migraine-like headache type (15, 16). As in previous reports on other coronaviruses, headache was an important symptom; we hypothesized the same for COVID-19 and intended to explore if it carries a prognostic value for early detection of the disease.

The prevalence of headache in adult COVID-19 patients is contradictory and inconclusive. A comprehensive meta-analysis can resolve the debate and aid in clinical diagnosis avoiding unnecessary delay in addition to managing COVID-19 patients in a more appropriate manner. Therefore, the primary objective of this systematic review and meta-analysis was to estimate the overall pooled prevalence of headache in adult patients with COVID-19. The secondary aim was to look for any linkage between the presence of headache and disease severity.

## Methods

### Search Strategy and Selection Criteria

We conducted a systematic review and meta-analysis of the literature in accordance with the PRISMA guideline (17) to identify studies published within the first 4 months of the COVID-19 outbreak (from 1st December 2019 to 31st March 2020) that presented the prevalence of headache in adult (≥18 years) patients with COVID-19 worldwide. This study is registered with PROSPERO (registration number: CRD42020182529). There was no restriction on the study design; therefore, observational studies, clinical trials, and case series were included. PubMed, Scopus, ScienceDirect, and Google Scholar databases were searched until 3rd April 2020 without language restrictions. The following search terms were searched in PubMed database and were modified to suit other databases: COVID-19, COVID19, coronavirus, nCoV, SARS-CoV-2, SARS-CoV2, clinical, symptom, symptoms, characteristic, characteristics, feature, features, condition, conditions, comorbid, co-morbid, comorbidity, co-morbidity, comorbidities, co-morbidities, epidemiological, epidemiology, and headache. Details of the search strategy are in the Supplementary Material (Supplementary Table 1). In addition to the published studies, preprints were also included if data of interest were reported. Review articles, case reports, opinions, and perspectives were excluded. Data reported by news reports and press releases or data collected from websites or databases were not considered. To ensure a robust search procedure, references of the included studies were also reviewed. Duplicate studies were excluded by using EndNote X8 software. To identify eligible studies, articles of interest were screened based on the title and abstract followed by full text by four authors (MAI, SSA, SK, and TH) independently. Disagreements about inclusion were discussed with MAI and CC and resolved by consensus.

### Data Extraction

Data extraction was done by MAI and cross-checked independently by the other three authors (SSA, SK, and TH). Before data extraction, all non-English-language studies were translated into English using Google Translate and validated by a native speaker. From each eligible study, we extracted the following information into a predefined Excel spreadsheet: first author's last name, region (country, province/municipalities/special administrative regions/city) of the participants, data collection period, COVID-19 confirmation procedure, total number of COVID-19 patients, number of female COVID-19 patients, age, subgroups of COVID-19 patients, and prevalence of headache.

### Data Analysis

Random-effects model was used to obtain the pooled prevalence and 95% confidence intervals (CIs) of headache in adult patients with COVID-19. Risk ratio (RR) with 95% CI was used to estimate the risk of experiencing headache in different subgroups of COVID-19 patients. Heterogeneity between studies was assessed using the *I*^2^ statistic (*I*^2^ > 75% indicating substantial heterogeneity) in addition to using the Cochran's *Q*-test to identify the significance of heterogeneity. Headache prevalence was also analyzed in different COVID-19 subgroups. All the analyses and plots were generated by using metaprop codes in meta (version 4.11-0) and metafor (version 2.4-0) packages of R (version 3.6.3) in RStudio (version 1.2.5033) and RevMan (version 5.3) software (18, 19).

### Study Quality Assessment

The quality of included studies was assessed independently by two authors (SSA and SK) using the Joanna Briggs Institute critical appraisal tools for cross-sectional, cohort, case series, randomized controlled trials, and case–control studies (20). Further, two authors (MAI and TH) validated the results of the quality assessment. The studies were classified as low quality (high risk of bias), moderate quality (moderate risk of bias), and high quality (low risk of bias) if the overall score was ≤49, 50–69, and ≥70%, respectively (21).

### Publication Bias

To assess publication bias, a funnel plot presenting prevalence estimates against their sample size was constructed and the asymmetry of the funnel plot was confirmed with Egger's test when a minimum of 10 studies was available.

### Sensitivity Analyses

To identify the source of heterogeneity and to check the robustness of the results, sensitivity analyses were performed through the following strategies: (i) excluding small studies (*n* < 100); (ii) excluding the low- and moderate-quality studies (high risk of bias); (iii) excluding studies without reporting the COVID-19 confirmation assay method; (iv) excluding non-English studies, (v) excluding outlier studies, and (vi) considering only cross-sectional studies. Additionally, to identify the outlier studies and the sources of heterogeneity, a Galbraith plot was constructed.

## Results

### Study Selection

**Figure 1:**
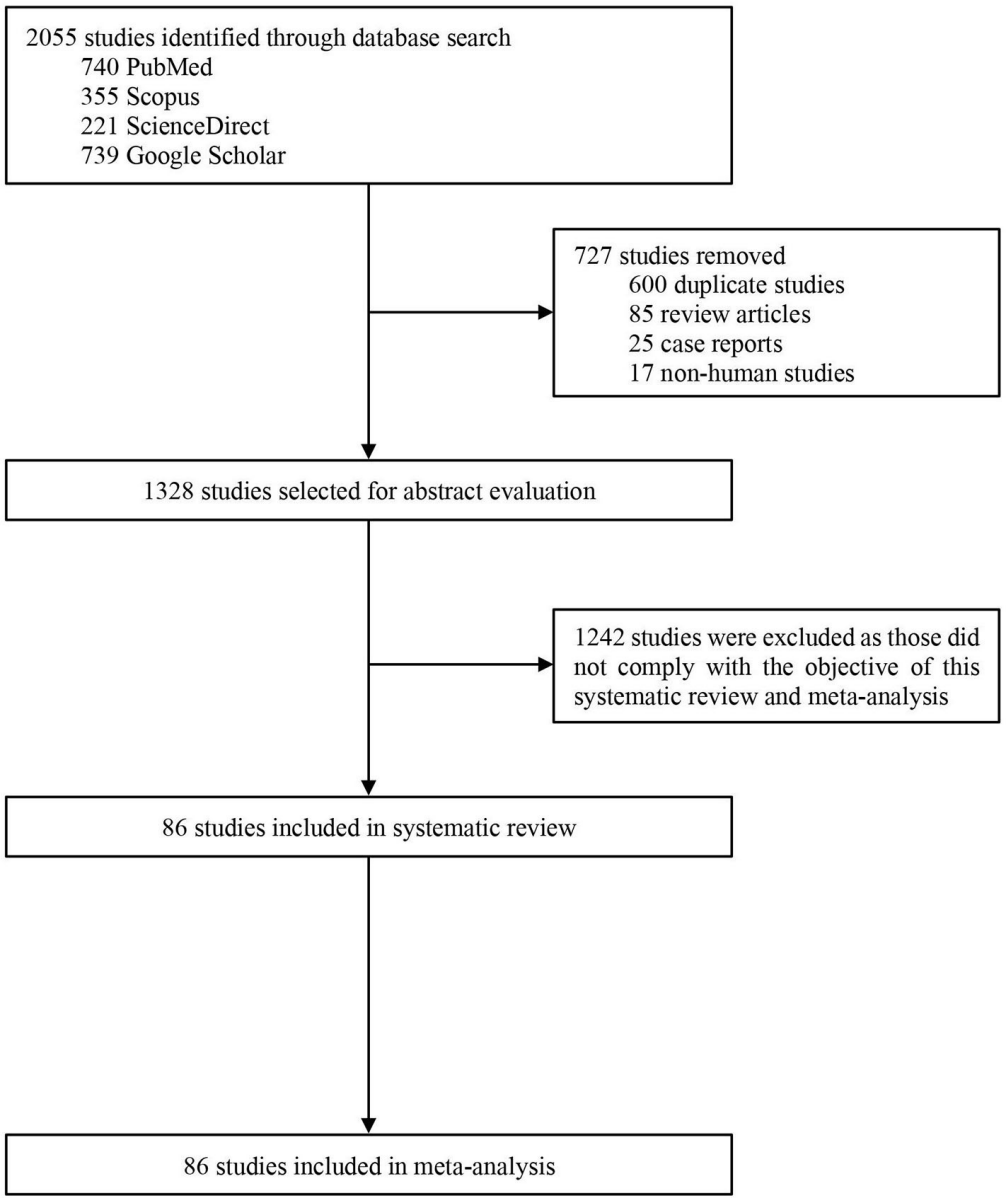
PRISMA flow diagram of study selection.

Our search initially identified 2,055 studies. After removing 727 studies [duplicate studies (*n* = 600), review articles (*n* = 85), case reports (*n* = 25), and non-human studies (*n* = 17)], titles and abstracts of 1,328 studies were screened for eligibility, of which 1,242 studies were excluded as those did not comply with the objective of this study. Therefore, 86 studies were included in the systematic review and meta-analysis (Figure 1).

### Characteristics of Included Studies

Detailed characteristics and references of the included studies are presented in the Supplementary Material (Supplementary Table 2). Overall, this meta-analysis reports data from 14,275 COVID-19 patients (49.4% female). Ages of the COVID-19 patients included in this meta-analysis ranged from 35.0 ± 8.0 to 70.7 ± 13.5 years. Studies were from two countries including China (85 studies, *n* = 14,251) and USA (one study, *n* = 24). Among the included studies, 91.9% confirmed COVID-19 patients by using the RT-PCR method, whereas method was not reported in 8.1% of the studies.

### Meta-Analysis

**Figure 2:**
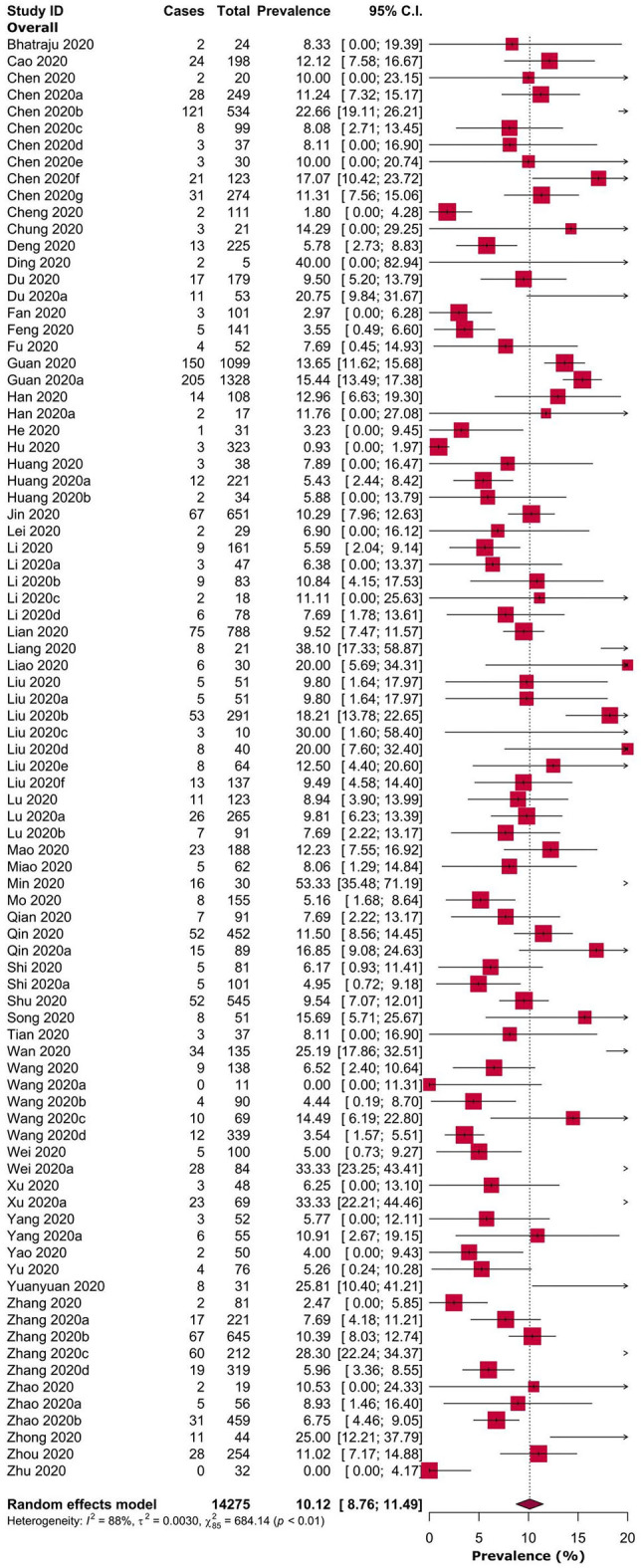
Prevalence of headache in adult COVID-19 patients.

Overall, the pooled prevalence of headache in COVID-19 patients was 10.1% [8.76–11.49] (Table 1, Figure 2). Prevalence of headache in Chinese and American patients were 10.1% [8.78–11.54] and 8.3% [0.00–19.39], respectively (Table 1 and Supplementary Figure 1). Headache prevalence in COVID-19 patients ranged between 3.1% [0.00–12.55] and 16.1% [8.01–24.21] in 13 Chinese provinces or municipalities (Table 1 and Supplementary Figure 2).

**Table 1:** Pooled prevalence of headache in COVID-19 patients from different regions.

**Regions**		**Headache prevalence [95% CI] (%)**	**Number of studies analyzed**	**Total number of COVID-19 patients**	**Heterogeneity**		**Publication bias, Egger's test (*p-*value)**
					***I^**2**^***	***p-*value**	
Overall		10.1 [8.76–11.49]	86	14,275	88%	<0.0001	0.40
China		10.1 [8.78–11.54]	85	14,115	88%	<0.0001	0.38
China provinces/municipalities	Hubei	9.5 [7.73–11.39]	48	6,578	88%	<0.0001	0.87
	Shanghai	11.0 [9.13–12.99]	6	1,013	0%	0.77	NA
	Zhejiang	9.3 [7.56–11.07]	5	2,553	52%	0.08	NA
	Beijing	10.1 [3.69–16.55]	5	217	60%	0.03	NA
	Chongqing	16.1 [8.01–24.21]	4	299	72%	0.01	NA
	Guangdong	9.6 [0.00–19.53]	3	380	85%	0.004	NA
	Anhui	3.1 [0.00–12.55]	2	51	51%	0.22	NA
	Hunan	5.1 [0.32–9.91]	2	197	42%	0.19	NA
	Shandong	14.0 [1.64–26.37]	2	90	68%	0.07	NA
	Jiangsu	5.4 [2.44–8.42]	1	221	NA	NA	NA
	Sichuan	7.6 [0.45–14.93]	1	52	NA	NA	NA
	Hebei	8.1 [0.00–16.90]	1	37	NA	NA	NA
	Hainan	9.8 [1.64–17.97]	1	51	NA	NA	NA
USA		8.3 [0.0–19.39]	1	24	NA	NA	NA

**Figure 3:**
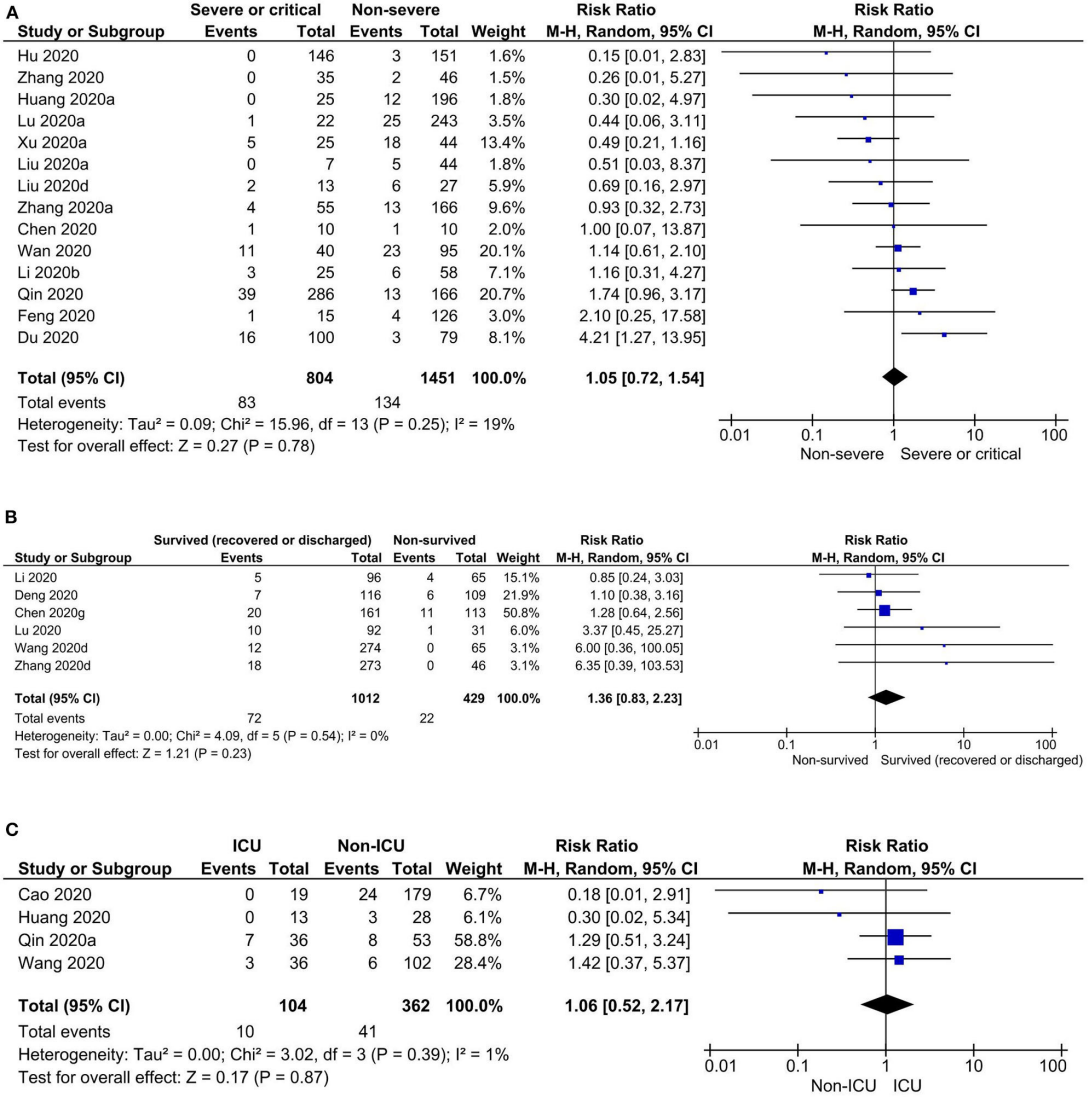
Risks of headache in **(A)** severe or critical vs. non-severe, **(B)** survived (recovered or discharged) vs. non-survived, and **(C)** ICU vs. non-ICU COVID-19 patients.

Risk of headache was observed to be higher in severe or critical COVID-19 patients when compared to non-severe COVID-19 patients, but not statistically significant (prevalence: 7.4 vs. 8.6%; RR: 1.05, 95% CI: 0.72–1.54; *p* = 0.78). Similarly, there were no significant differences in risk of headache in survived (recovered or discharged) vs. non-survived (prevalence: 7.1 vs. 3.3%; RR: 1.36, 95% CI: 0.83–2.23; *p* = 0.23) and ICU vs. non-ICU COVID-19 patients (prevalence: 5.8 vs. 10.6%; RR: 1.06, 95% CI: 0.52–2.17; *p* = 0.87) (Table 2, Figure 3, and Supplementary Figure 3). In pregnant women, the prevalence of headache was 6.4% [0.00–15.10] (Table 2). Overall, diverse levels of heterogeneity were observed during estimation of the prevalence of headache in COVID-19 patients from different regions (ranging from 0 to 88%) (Table 1) as well as different subgroups (ranging from 29 to 82%) (Table 2).

**Table 2:** Pooled prevalence of headache in different subgroups of adult COVID-19 patients.

**Subgroups of adult COVID-19 patients**	**Headache prevalence [95% CIs] (%)**	**Number of studies analyzed**	**Total number of COVID-19 patients**	**Heterogeneity**		**Publication bias, Egger's test (*p*-value)**
				***I^**2**^***	***p-*value**	
Severe or critical	7.4 [3.93–10.87]	19	975	82%	<0.0001	0.50
Non-severe	8.6 [5.74–11.51]	15	1,551	80%	<0.0001	0.20
Survived (recovered or discharged)	7.1 [5.30–8.99]	11	1,215	29%	0.17	0.14
Non-survived	3.3 [0.78–5.83]	7	530	67%	0.03	NA
ICU patients	5.8 [0.00–13.62]	4	104	64%	0.12	NA
Non-ICU patients	10.6 [5.81–15.46]	4	362	50%	0.11	NA
Pregnant women	6.4 [0.0–15.10]	1	31	NA	NA	NA

### Study Quality Assessment

Detailed quality assessment of the included studies is shown in the Supplementary Materials (Supplementary Tables 3–7). Briefly, 64.0, 34.9, and 1.1% of the included studies were of high-, moderate-, and low-quality studies, of which a single cross-sectional study was of low quality (high risk of bias).

### Publication Bias

**Figure 4:**
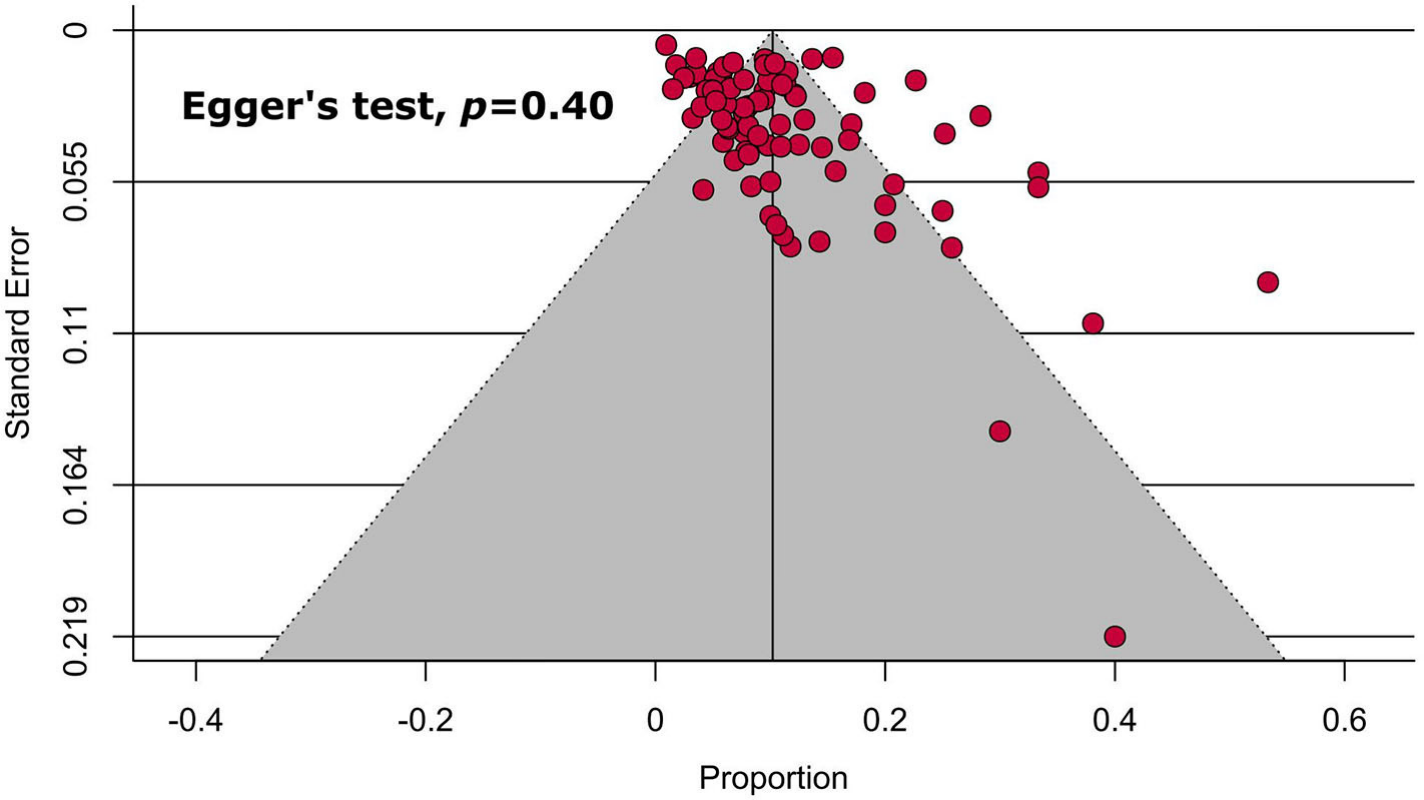
Funnel plot on the prevalence of headache in COVID-19 patients.

Following visual inspection and Egger's test results, none of the main (Table 1, Figure 4) and subgroup analyses (Table 2) exhibited significant publication bias.

### Sensitivity Analyses

Sensitivity analyses on assessing headache in COVID-19 patients excluding studies on the basis of small studies, low- and medium-quality studies, COVID-19 confirmation test assay not being reported, non-English studies, outlier studies, and considering only cross-sectional studies showed marginal differences in overall pooled prevalence ranging from 12.6% lower to 0.7% higher (Table 3 and Supplementary Figure 4). Overall, our sensitivity analyses indicated that the results of headache prevalence in COVID-19 patients are reliable and robust. As the sources of heterogeneity, although we identified seven outlier studies from the Galbraith plot (Supplementary Figure 5), performing a sensitivity analysis excluding these outlier studies could not reduce the levels of heterogeneity.

**Table 3:** Sensitivity analyses.

**Strategies of sensitivity analyses**	**Fever prevalence [95% CIs] (%)**	**Difference of pooled prevalence compared to the main result**	**Number of studies analyzed**	**Total number of COVID-19 patients**	**Heterogeneity**	
					***I^**2**^***	***p*-value**
Excluding small studies	9.7 [7.90–11.62]	3.6% lower	37	11,893	93%	<0.0001
Excluding low- and moderate-quality studies	10.1 [8.3–11.9]	0.7% higher	55	10,551	90%	<0.0001
Excluding studies without reporting COVID-19 confirmation method	9.9 [8.58–11.37]	1.4% lower	79	13,987	88%	<0.0001
Excluding non-english studies	9.8 [8.50–11.21]	2.6% lower	83	14,185	87%	<0.0001
Excluding outlier studies	8.8 [7.57–10.10]	12.6% lower	79	13,693	85%	<0.0001
Considering only cross-sectional studies	10.0 [8.65–11.50]	0.4% lower	74	12,193	87%	<0.0001

## Discussion

### Summary of Evidence

Based on the findings of this meta-analysis, headache was estimated to be in 10.1% of the adult COVID-19 patients. The prevalence of headache in COVID-19 is less common than SARS (20.0–61.0%) (22, 23) and MERS (12.9–23.0%) (24, 25) and even five times lesser compared to the prevalence of headache in the general population (50%) (26). Headache is observed in over 90 and 60% of patients with influenza and acute upper respiratory tract viral infections (27, 28). Compared to the results of our meta-analysis, headache prevalence in severe or critical COVID-19 patients was almost half of that in severe or critical MERS patients (15.6%) (29) and ~4-fold higher in severe SARS patients (30). Similar to our findings, risk of headache was observed high in survived patients compared to non-survived patients with MERS (29, 31). The prevalence of headache in ICU MERS patients was ~7.5 times higher than that in COVID-19 (32). In pregnant women with SARS, the prevalence of headache was ~9 times higher compared to our findings in pregnant COVID-19 patients (33); however, we were able to analyze only a single study on pregnant women, and hence, this result should be considered with caution. No significant difference of risk of headache was observed between (i) severe or critical vs. non-severe, (ii) survived (recovered or discharged) vs. non-survived, and (iii) ICU vs. non-ICU COVID-19 patients, and these analyses were done based on the available data from only 14, 6, and 4 studies, respectively; therefore, this should not be considered as a conclusive result.

### Strengths

Our study has several strengths. This meta-analysis is the first, to our knowledge, to comprehensively investigate the prevalence of headache in adult and different subgroups of COVID-19 patients. This meta-analysis was conducted with a large number of studies and hence including a large number of participants, resulting in more robust estimates. We included both English and non-English-language articles, and the non-English-language articles do not seem to affect overall estimates in this meta-analysis. Majority of the included studies confirmed COVID-19 subjects by using the RT-PCR technique, which strengthens our findings. None of the analyses represented significant publication bias, demonstrating that we were unlikely to have missed studies that could have altered the findings. The major sources of heterogeneity were identified by the Galbraith plot. All the conducted sensitivity analyses generated similar results to the main findings, indicating the robustness of the meta-analysis results.

### Limitations

Nevertheless, there are several notable limitations. Based on the search strategy and considered time period, this meta-analysis could include only one study conducted outside China; therefore, the prevalence may not represent at a global scale and generalization of the findings should be done with care. Most of the analyses generated substantial degrees of heterogeneity. Even though we examined the sources of heterogeneity by subgroup, sensitivity analyses, and Galbraith plot, heterogeneity could not be fully explained by the factors included in the analyses. Based on the quality assessment of the included studies, 36% of the studies were low- and moderate-quality studies; excluding these studies though generated almost identical results of the main findings; however, the overall headache prevalence should be considered with caution. Though we identified the prevalence of headache from the first 4-month data of the COVID-19 outbreak, we were unable to characterize headache type due to lack of information. Therefore, in the future, type of headache in COVID-19 patients could be interesting to explore.

### Implications for Further Research

From the first 4-month data, even though we estimated low prevalence of headache in COVID-19 patients, it would be interesting to conduct meta-analyses on data from April 2020 and beyond, so that besides the study from China, the prevalence of headache can be detected in COVID-19 patients from other countries. Studies on the mechanism of CNS involvement in patients with COVID-19 have been carried out, and the main mechanism for damage was found to be inflammation—causing blood–brain barrier deterioration and inflammation of endothelial cells in vascular and cerebral tissues (34). These can explain the main migraine-like characteristics of some description in the literature (14–16). More should however be explained, possibly through larger studies on headache characteristics together with other parameters on inflammation, thrombophilic alteration, brain histology in autopsies, brain scan, and perfusion.

## Conclusions

We estimated the prevalence of headache reported during admission as 10.1% in adult COVID-19 patients. Based on the first 4-month data of the outbreak, headache was not observed as one of the most common initial symptoms in adult COVID-19 patients. Therefore, in addition to headache, other clinical manifestations should be considered. In conclusion, the findings from this meta-analysis represent the most comprehensive and robust currently available evidence of headache prevalence in adult COVID-19 patients. We hope that these results will assist in the decision making of patients, clinicians, and policy makers.

## Data Availability Statement

The original contributions presented in the study are included in the article/Supplementary Materials, further inquiries can be directed to the corresponding author/s.

## Author Contributions

MI conceived the idea of the study, developed the protocol, extracted data and relevant information, undertook the statistical analyses, and drafted the manuscript. MI, SK, SA, and TH searched the literature and applied inclusion and exclusion criteria. SK and SA conducted quality assessment. Disagreements were resolved by consensus with MI and CC. All authors reviewed, edited, and approved the final version of the manuscript.

## Conflict of Interest

The authors declare that the research was conducted in the absence of any commercial or financial relationships that could be construed as a potential conflict of interest.
