# Supplementary material for: Prevalence of Headache in Patients With Coronavirus Disease 2019 (COVID-19): A Systematic Review and Meta-Analysis of 14,275 Patients
Source: Front Neurol. 2020 Nov 27;11:562634. doi: 10.3389/fneur.2020.562634 (PMC7728918; doi:10.3389/fneur.2020.562634)
Supplement: Supplementary file 1 [file Table_1.DOCX]

| **Supplementary Table 1. Search strategy** | |
| --- | --- |
| **Databases** | **Search terms** |
| PubMed | ((((((COVID-19[Title]) OR COVID19[Title]) OR coronavirus[Title]) OR nCoV[Title]) OR SARS-CoV-2[Title]) OR SARS-CoV2[Title])) AND (((((((((((((((((((clinical[Title/Abstract]) OR symptom[Title/Abstract]) OR symptoms[Title/Abstract]) OR characteristic[Title/Abstract]) OR characteristics[Title/Abstract]) OR feature[Title/Abstract]) OR features[Title/Abstract]) OR condition[Title/Abstract]) OR conditions[Title/Abstract]) OR comorbid[Title/Abstract]) OR co-morbid[Title/Abstract]) OR comorbidity[Title/Abstract]) OR co-morbidity[Title/Abstract]) OR comorbidities[Title/Abstract]) OR co-morbidities[Title/Abstract]) OR epidemiological[Title/Abstract]) OR epidemiology[Title/Abstract]) OR headache[Title/Abstract]) |
| Scopus | TITLE-ABS(COVID-19 OR COVID19 OR coronavirus OR nCoV OR SARS-CoV-2 OR SARS-CoV2) AND TITLE-ABS(clinical OR symptom OR symptoms OR characteristic OR characteristics OR feature OR features OR condition OR conditions OR comorbid OR co-morbid OR comorbidity OR co-morbidity OR comorbidities OR co-morbidities OR epidemiological OR epidemiology OR headache) AND ( LIMIT-TO ( PUBYEAR,2020) OR LIMIT-TO ( PUBYEAR,2019)) |
| ScienceDirect | Title, abstract, keywords: (COVID-19 OR COVID19 OR coronavirus OR nCoV OR SARS-CoV-2 OR SARS-CoV2) AND (clinical OR symptom OR symptoms OR characteristic OR characteristics OR feature OR features OR condition OR conditions OR comorbid OR co-morbid OR comorbidity OR co-morbidity OR comorbidities OR co-morbidities OR epidemiological OR epidemiology OR headache) |
| Google Scholar | allintitle:(COVID-19 OR COVID19 OR coronavirus OR nCoV OR SARS-CoV-2 OR SARS-CoV2) (clinical OR symptom OR symptoms OR characteristic OR characteristics OR feature OR features OR condition OR conditions OR comorbid OR co-morbid OR comorbidity OR co-morbidity OR comorbidities OR co-morbidities OR epidemiological OR epidemiology OR headache) |
