# Supplementary material for: Prevalence of Headache in Patients With Coronavirus Disease 2019 (COVID-19): A Systematic Review and Meta-Analysis of 14,275 Patients
Source: Front Neurol. 2020 Nov 27;11:562634. doi: 10.3389/fneur.2020.562634 (PMC7728918; doi:10.3389/fneur.2020.562634)
Supplement: Supplementary file 2 [file Table_2.DOCX]

| **Supplementary Table 2. Major characteristics of the included studies** | | | | | | | |  |
| --- | --- | --- | --- | --- | --- | --- | --- | --- |
| **No.** | **Study ID**  **(Reference)** | **Country**  **(province/** **municipalities/** **special administrative regions / city)** | **Data collection period** | **COVID-19 confirmation procedure** | **Total number of COVID-19 patients (female)** | **Age (years) (mean±SD / median (IQR) / range** | **Subgroups of COVID-19 patients** | |
| 1 | Bhatraju 2020  (1) | USA  (Seattle) | 24 Feb to 9 Mar, 2020 | RT-PCR | 24 (9) | 64·0±18·0 | All critical ICU patients | |
| 2 | Cao 2020  (2) | China  (Shanghai) | 20 Jan to 15 Feb, 2020 | RT-PCR | 198 (97) | 50·1±16·3 | ICU (n=19) and non-ICU (n=179) | |
| 3 | Chen 2020  (3) | China  (Hubei) | 19 Dec, 2019 to 27 Jan, 2020 | RT-PCR | 21 (4) | 56·3±14·3 | Severe (n=11) and non-severe (n=10) | |
| 4 | Chen 2020a  (4) | China  (Shanghai) | 20 Jan to 6 Feb, 2020 | RT-PCR | 249 (123) | 51·0 (36·0-64·0) | ICU (n=22) and non-ICU (n=227) | |
| 5 | Chen 2020b  (5) | China  (Hubei) | 1 Feb to 1 Mar, 2020 | RT-PCR | 534 (266) | Mobile cabin hospital: 40·0 (16·0-68·0); hospital: 50·0 (19·0-65·0) | Mobile cabin hospital (n=263) and hospital (n=271) | |
| 6 | Chen 2020c  (6) | China  (Hubei) | 1 Jan to 20 Jan, 2020 | RT-PCR | 99 (32) | 55·5±13·1 | None | |
| 7 | Chen 2020d  (7) | China  (Hebei) | 22 Jan to 14 Feb, 2020 | RT-PCR | 37 (16) | 58·7±13·7 | All severe or critical patients | |
| 8 | Chen 2020e  (8) | China  (Hubei) | Jan to Mar, 2020 | RT-PCR | 30 (16) | 60·5 (32·0-77·0) | None | |
| 9 | Chen 2020f  (9) | China  (Hubei) | 5 Jan to 7 Mar, 2020 | RT-PCR | 123 (73) | 51·0 (35·0-66·0) | With HBV  infection  (n=15) and  without HBV  infection  (n=108) | |
| 10 | Chen 2020g  (10) | China  (Hubei) | 13 Jan to 12 Feb, 2020 | RT-PCR | 274 (103) | 62·0 (44·0-70·0) | Survived (n=161) and non-survived (n=274) | |
| 11 | Cheng 2020  (11) | China  (Hubei) | 15 Jan to 23 Feb, 2020 | RT-PCR | 111 (111) | 32·0 (22·0-41·0) | Pregnant  (n=31) and  non-pregnant  (n=80) | |
| 12 | Chung 2020  (12) | China  (Guangdong, Jiangxi and Shandong) | 18 Jan to 27 Jan, 2020 | NR | 21 (8) | 51·0±41·0 | None | |
| 13 | Deng 2020  (13) | China  (Hubei) | 1 Jan to 21 Feb, 2020 | RT-PCR | 225 (101) | Non-survived: 69·0 (62·0-74·0) and survived 40·0 (33·0-57·0) | Survived (n=116) and non-survived (n=109) | |
| 14 | Ding 2020  (14) | China  (Hubei) | NR | NR | 5 (3) | 39·0-66·0 | None | |
| 15 | Du 2020  (15) | China  (Hubei) | 25 Dec to 7  Feb, 2020 | RT-PCR | 179 (82) | 57·6±13·7 | Severe or critical (n=100) and non-severe (n=79) | |
| 16 | Du 2020a  (16) | China  (Shandong) | 23 Jan to 25  Feb, 2020 | RT-PCR | 67 (35) | Adult: 41·4 (median); paediatric: 6·2 (median) | Severe (n=1) and non-severe (n=66) | |
| 17 | Fan 2020  (17) | China  (Hubei) | 30 Dec to 16  Feb, 2020 | RT-PCR | 101 (37) | 65·4±9·7 | All non-survived patients | |
| 18 | Feng 2020  (18) | China  (Hunan) | 17 Jan to 1  Feb, 2020 | RT-PCR | 141 (69) | 44·0 (34·0-55·0) | Severe (n=15) and non-severe (n=126) | |
| 19 | Fu 2020  (19) | China  (Sichuan) | 1 Jan to 20 Feb, 2020 | RT-PCR | 52 (24) | 44·5 (33·0-56·5) | Severe or critical (n=14) and non-severe (n=38) | |
| 20 | Guan 2020  (20) | China  (30 provinces / autonomous regions / provincial municipalities) | 11 Dec to 29  Jan, 2020 | RT-PCR | 1096 (459) | 47·0 (35·0-58·0) | Severe (n=173) and non-severe (n=926) | |
| 21 | Guan 2020a  (21) | China  (31 provinces / autonomous regions / provincial municipalities) | 21 Nov, 2019 to 31 Jan, 2020 | RT-PCR | 1590 (686) | 48·9±16·3 | With comorbidity (n=399) or without comorbidity (n=1191) | |
| 22 | Han 2020  (22) | China  (Hubei) | 4 Jan to 3  Feb, 2020 | RT-PCR | 108 (70) | 21·0-90·0 | None | |
| 23 | Han 2020a  (23) | China  (Hubei) | 20 Dec to 2  Feb, 2020 | RT-PCR | 17 (11) | 40·0±10·0 | None | |
| 24 | He 2020  (24) | China  (Hubei) | 23 Jan to 14 Feb, 2020 | RT-PCR | 31 (18) | 34·0 (25·5-47·0) | With HDs (n=15) and without HDs (n=16) | |
| 25 | Hu 2020  (25) | China  (Hubei) | 8 Jan to 10 March, 2020 | RT-PCR | 323 (157) | 61·0 (23·0-91·0) | Severe (n=151) and non-severe or critical (n=172) | |
| 26 | Huang 2020  (26) | China  (Hubei) | 16 Dec to 2  Jan, 2020 | RT-PCR | 41 (11) | 49·0 (41·0-58·0) | ICU (n=13) and non-ICU (n=28) | |
| 27 | Huang 2020a  (27) | China  (Jiangsu) | 22 Jan to 10 February, 2020 | RT-PCR | 221 (95) | 45·0 (33·5-56·0) | Severe or critical (n=25) and non-severe (n=196) | |
| 28 | Huang 2020b  (28) | China  (Hubei) | 21 Dec to 28 Jan, 2020 | RT-PCR | 34 (20) | 56·2±17·1 | None | |
| 29 | Jin 2020  (29) | China  (Zhejiang) | 17 Jan to 8  Feb, 2020 | RT-PCR | 651 (320) | 45·6±14·3 | With GI symptoms (n=74) and without GI symptoms (n=577) | |
| 30 | Lei 2020  (30) | China  (Hubei) | 14 Jan to 29 Jan, 2020 | NR | 29 (8) | 26·0-79·0 | Severe or critical (n=14) and non-severe (n=15) | |
| 31 | Li 2020  (31) | China  (Hubei) | 21 Jan to 14 Feb, 2020 | RT-PCR | 161 (58) | Survived: 46·0 (22·0-87·0) and non-survived 67·0 (31·0-87·0) | Survived (n=96) and non-survived (n=65) | |
| 32 | Li 2020a  (32) | China  (Beijing) | 8 Feb to 22 Feb, 2020 | RT-PCR | 47 (19) | 62·0 (51·0-70·0) | All severe patients | |
| 33 | Li 2020b  (33) | China  (Chongqing) | 1 Jan to 29 Feb, 2020 | RT-PCR | 83 (39) | 45·5±12·3 | Severe or critical (n=25) and non-severe (n=58) | |
| 34 | Li 2020c  (34) | China  (Beijing) | 19 Jan to 22 Feb, 2020 | RT-PCR | 18 (7) | 39·0 (34·0-60·2) | None | |
| 35 | Li 2020d  (35) | China  (Guangdong) | 18 Jan to 7 Feb, 2020 | RT-PCR | 78 (40) | 44·6±17·9 | None | |
| 36 | Lian 2020  (36) | China  (Zhejiang) | 17 Jan to 12 Feb, 2020 | RT-PCR | 788 (381) | 54·7±9·3 | Older (≥ 60 years) (n=136) and younger (< 60 years) (n=652) | |
| 37 | Liang 2020  (37) | China  (Beijing) | 21 Jan to 15 Feb, 2020 | RT-PCR | 21 (10) | 42·0 (34·5-66·0) | None | |
| 38 | Liao 2020  (38) | China  (Chongqing) | 25 Jan to 18 Feb, 2020 | RT-PCR | 32 (15) | 25·0-35·0 | None | |
| 39 | Liu 2020  (39) | China  (Hainan) | 1 Jan to 16 Feb, 2020 | NR | 51 (23) | 50·4±13·0 | None | |
| 40 | Liu 2020a  (40) | China  (Chongqing) | 20 Jan to 3 Feb, 2020 | NR | 51 (19) | 45·0 (34·0-51·0) | Severe (n=7) and non-severe (n=44) | |
| 41 | Liu 2020b  (41) | China  (Guangdong) | 10 Jan to 24 Feb, 2020 | RT-PCR | 291 (158) | 48·1 (34·0-62·0) | With cardiac injury (n=15) and without cardiac injury (n=276) | |
| 42 | Liu 2020c  (42) | China  (Zhejiang) | 22 Jan to 11  Feb, 2020 | RT-PCR | 10 (6) | 43·0±10·4 | None | |
| 43 | Liu 2020d  (43) | China  (Hubei) | 5 Jan to 24 Jan, 2020 | RT-PCR | 40 (25) | 48·7±13·9 | Severe (n=13) and non-severe (n=27) | |
| 44 | Liu 2020e  (44) | China  (Hubei) | 16 Jan to 15 Feb, 2020 | RT-PCR | 64 (41) | 35·0 (29·0-43·0) | Symptoms onset ≤10 days (n=37) and symptoms onset >10 days (n=25) | |
| 45 | Liu 2020f  (45) | China  (Hubei) | 30 Dec, 2019 to 24 Jan, 2020 | RT-PCR | 137 (76) | 57·0 (20·0-83·0) | None | |
| 46 | Lu 2020  (46) | China  (Hubei) | 1 Jan to 15 Feb, 2020 | RT-PCR | 123 (62) | 62·5±11·5 | Survived (n=92) and non-survived (n=31) | |
| 47 | Lu 2020a  (47) | China  (Shanghai) | 20 Jan to 19 Feb, 2020 | RT-PCR | 265 (NR) | NR | Severe or critical (n=243) and non-severe (n=22) | |
| 48 | Lu 2020b  (48) | China  (Hubei) | 21 Jan to 10 Feb, 2020 | RT-PCR | 91 (42) | 47·6±15·6 | None | |
| 49 | Mao 2020  (49) | China  (Shanghai) | 17 Jan to 16 Feb, 2020 | RT-PCR | 188 (94) | 46·7±16·1 | None | |
| 50 | Miao 2020  (50) | China  (Shanghai) | 12 Jan to 13 Feb, 2020 | RT-PCR | 62 (30) | 43·8±13·9 | None | |
| 51 | Min 2020  (51) | China  (Hubei) | 3 Jan to 11 Jan, 2020 | NR | 30 (20) | 35·0±8·0 | Severe (n=22) and non-severe (n=243) | |
| 52 | Mo 2020  (52) | China  (Hubei) | 1 Jan to 5 Feb, 2020 | NAT | 155 (69) | 54·0 (42·0-66·0) | General (n=70) and refractory (n=85) | |
| 53 | Qian 2020  (53) | China  (Hubei) | 20 Jan to 11 Feb, 2020 | RT-PCR | 91 (54) | 50·0 (36·5-57·0) | Severe (n=82) and non-severe (n=9) | |
| 54 | Qin 2020  (54) | China  (Hubei) | 10 Jan to 12 Feb, 2020 | RT-PCR | 452 (217) | 58·0 (47·0-67·0) | Severe (n=286) and non-severe (n=166) | |
| 55 | Qin 2020a  (55) | China  (Hubei) | 23 Jan to 11 Feb, 2020 | RT-PCR | 89 (43) | 55·0 (23·0-86·0) | ICU (n=35) and non-ICU (n=53) | |
| 56 | Shi 2020  (56) | China  (Hubei) | 20 Dec to 23 Jan, 2020 | RT-PCR | 81 (39) | 49·5±11·0 | NR | |
| 57 | Shi 2020a  (57) | China  (Hubei) | Until 15 Feb, 2020 | NR | 101 (41) | 71·0 (59·0-80·0) | All non-survived patients | |
| 58 | Shu 2020  (58) | China  (Hubei) | 13 Feb to 29 Feb, 2020 | RT-PCR | 545 (281) | 50·0 (38·0- 58·0) | None | |
| 59 | Song 2020  (59) | China  (Shanghai) | 20 Jan to 27 Jan, 2020 | RT-PCR | 51 (26) | 49·6±16·0 | None | |
| 60 | Tian 2020  (60) | China  (Shandong) | NR | RT-PCR | 37 (20) | 44·3±1·6 | Quarantine onset duration ≤20 days (n=19) and >20 days (n=18) | |
| 61 | Wan 2020  (61) | China  (Chongqing) | 23 Jan to 8 Feb, 2020 | RT-PCR | 135 (63) | 47·0 (36·0-55·0) | Severe (n=40) and non-severe (n=95) | |
| 62 | Wang 2020  (62) | China  (Hubei) | 1 Jan to 3 Feb, 2020 | RT-PCR | 138 (63) | 56·0 (42·0-68·0) | ICU (n=36) and non-ICU (n=102) | |
| 63 | Wang 2020a  (63) | China  (Guangdong) | 26 Jan to 19 Feb, 2020 | RT-PCR | 11 (1) | 58·0 (49·0-72·0) | All severe or critical patients | |
| 64 | Wang 2020b  (64) | China  (Hubei) | 16 Jan to 17 Feb, 2020 | RT-PCR | 90 (57) | 45·0±14·0 | None | |
| 65 | Wang 2020c  (65) | China  (Hubei) | 16 Jan to 29 Jan, 2020 | RT-PCR | 69 (37) | 42·0 (35·0-62·0) | SpO_2_ ≥90% (n=55) and  SpO_2_ <90% (n=14) | |
| 66 | Wang 2020d  (66) | China  (Hubei) | 1 Jan to 6 Feb, 2020 | RT-PCR | 339 (173) | 69·0 (65·0-76·0) | Survived (n=174) and non-survived (n=65) | |
| 67 | Wei 2020  (67) | China  (Hubei) | 1 Feb to 28 Feb, 2020 | RT-PCR | 100 (60) | 49·1±17·2 | All non-severe patients | |
| 68 | Wei 2020a  (68) | China  (Hubei) | 19 Jan to 7 Feb, 2020 | RT-PCR | 84 (56) | 37·0 (24·0-74·0) | Diarrhoea group  (n=26)  non-diarrhoea  group  (n=58) | |
| 69 | Xu 2020  (69) | China  (Hubei) | 6 Jan to 31 Jan, 2020 | RT-PCR | 48 (35) | 39·0±13·8 | Non-hospitalized  patients (n=42) and  hospitalized  patients (n=6) | |
| 70 | Xu 2020a  (70) | China  (Hubei, Beijing and Shanghai) | 7 Feb to 28 Feb, 2020 | RT-PCR | 69 (34) | 57·0 (43·0-69·0) | Severe or critical (n=25) and non-severe (n=44) | |
| 71 | Yang 2020  (71) | China  (Hubei) | 24 Dec, 2019 to 26 Jan, 2020 | RT-PCR | 52 (17) | 59·7±13·3 | All severe or critical patients, survived  (n=20) and  non-survived  (n=32) | |
| 72 | Yang 2020a  (72) | China  (Beijing) | 27 Dec, 2019 to 18 Feb, 2020 | RT-PCR | 55 (22) | 44·0 (34·0-54·0) | Severe (n=14) and non-severe (n=41) | |
| 73 | Yao 2020  (73) | China  (Hubei) | 26 Jan to 18 Feb, 2020 | RT-PCR | 55 (18) | 70·7±13·5 | None | |
| 74 | Yu 2020  (74) | China  (Beijing) | 5 Feb to 19 Feb, 2020 | RT-PCR | 76 (38) | 40·0 (32·0-63·0) | None | |
| 75 | Yuanyuan 2020  (75) | China  (Hubei) | Jan to Feb, 2020 | RT-PCR | 31 (16) | 54·0±13·0 | Severe (n=11) and non-severe (n=20) | |
| 76 | Zhang 2020  (76) | China  (Hubei) | 30 Jan to 15 Feb, 2020 | RT-PCR | 81 (36) | NR | Severe or critical (n=35) and non-severe (n=46) | |
| 77 | Zhang 2020a  (77) | China  (Hubei) | 2 Jan to 10 Feb, 2020 | RT-PCR | 221 (113) | 55·0 (39·0-66·5) | Severe (n=55) and non-severe (n=166) | |
| 78 | Zhang 2020b  (78) | China  (Zhejiang) | 17 Jan to 8 Feb, 2020 | NAT | 645 (317) | 40·8±14·0 | Normal imaging findings (n=72) and  abnormal imaging findings (n=573) | |
| 79 | Zhang 2020c  (79) | China  (Hubei) | 18 Feb to 12 Mar, 2020 | NAT | 212 (127) | 48·5±13·1 | None | |
| 80 | Zhang 2020d  (80) | China  (Hubei) | 2 Jan to 13 Feb, 2020 | RT-PCR | 319 (171) | 47·0 (34·0-61·0) | Survived (n=273) and non-survived (n=46) | |
| 81 | Zhao 2020  (81) | China  (Anhui) | 23 Jan to 5 Feb, 2020 | RT-PCR | 19 (8) | 48·0 (27·0-56·0) | None | |
| 82 | Zhao 2020a  (82) | China  (Hunan) | 16 Jan to 6 Feb, 2020 | RT-PCR | 56 (30) | 50·3±15·6 | None | |
| 83 | Zhao 2020b  (83) | China  (Zhejiang) | 27 Jan to 12 Feb, 2020 | RT-PCR | 459 (215) | 48·0 (2·0-93·0) | Severe (n=42) and non-severe (n=417) | |
| 84 | Zhong 2020  (84) | China  (Hubei) | 1 Jan to 14 Feb, 2020 | RT-PCR | 49 (42) | 31·0 (29·0-34·0) | None | |
| 85 | Zhou 2020  (85) | China  (Hubei) | 20 Dec, 2019 to 9 Feb, 2020 | NAT | 254 (139) | 50·0 (36·0-65·0) | Medical staff (n=93) and  non-medical staff (n=161) | |
| 86 | Zhu 2020  (86) | China  (Anhui) | 24 Jan to 20 Feb, 2020 | NAT | 32 (17) | 46·0 (35·0‐52·0) | None | |
| SD: Standard deviation; IQR: interquartile range; RT-PCR: reverse transcription polymerase chain reaction; ICU: intensive care unit; NAT: nucleic acid test; HDs: haematological disorders; NR: not reported. | | | | | | | |  |

**References**

1. Bhatraju PK, Ghassemieh BJ, Nichols M, Kim R, Jerome KR, Nalla AK, et al. Covid-19 in critically ill patients in the Seattle region-case series. *N Engl J Med.* (2020) <https://doi.org/10.1056/NEJMoa2004500>.

2. Cao M, Zhang D, Wang Y, Lu Y, Zhu X, Li Y, et al. Clinical features of patients infected with the 2019 novel coronavirus (COVID-19) in Shanghai, China. *medRxiv.* (2020) <https://doi.org/10.1101/2020.03.04.20030395>.

3. Chen G, Wu D, Guo W, Cao Y, Huang D, Wang H, et al. Clinical and immunologic features in severe and moderate forms of Coronavirus Disease 2019. *medRxiv.* (2020) <https://doi.org/10.1101/2020.02.16.20023903>.

4. Chen J, Qi T, Liu L, Ling Y, Qian Z, Li T, et al. Clinical progression of patients with COVID-19 in Shanghai, China. *J Infect.* (2020) 80:1-6.

5. Chen L, Deng C, Chen X, Zhang X, Chen B, Yu H, et al. Ocular manifestations and clinical characteristics of 534 cases of COVID-19 in China: A cross-sectional study. *medRxiv.* (2020) <https://doi.org/10.1101/2020.03.12.20034678>.

6. Chen N, Zhou M, Dong X, Qu J, Gong F, Han Y, et al. Epidemiological and clinical characteristics of 99 cases of 2019 novel coronavirus pneumonia in Wuhan, China: a descriptive study. *Lancet.* (2020) 395:507-13.

7. Chen Y, Zhang K, Zhu G, Liu L, Yan X, Cai Z, et al. Clinical Characteristics and Current Treatment of Critically Ill Patients with COVID-19 Outside Wuhan, China: A Multicenter, Retrospective, Observational Study. *SSRN.* (2020) <https://ssrn.com/abstract=3551426>.

8. Chen X, Yuan W, Shao Z, Liu G, Wang W, Zhang S, et al. The Desynchrony between Clinical Course and RT-PCR Test Results in Patients with COVID-19 Infected Pneumonia During the Treatment in Wuhan, China. *SSRN.* (2020) <https://ssrn.com/abstract=3551404>.

9. Chen X, Jiang Q, Ma Z, Ling J, Hu W, Cao Q, et al. Clinical Characteristics of Hospitalized Patients with SARS-CoV-2 and Hepatitis B virus Co-infection. *medRxiv.* (2020) <https://doi.org/10.1101/2020.03.23.20040733>.

10. Chen T, Wu D, Chen H, Yan W, Yang D, Chen G, et al. Clinical characteristics of 113 deceased patients with coronavirus disease 2019: retrospective study. *BMJ.* (2020) 368:<https://doi.org/10.1136/bmj.m091>.

11. Cheng B, Jiang T, Zhang L, Hu R, Tian J, Jiang Y, et al. Clinical Characteristics of Pregnant Women with Coronavirus Disease 2019 in Wuhan, China. *SSRN.* (2020) <https://ssrn.com/abstract=3555240>.

12. Chung M, Bernheim A, Mei X, Zhang N, Huang M, Zeng X, et al. CT imaging features of 2019 novel coronavirus (2019-nCoV). *Radiology.* (2020) 295:202-7.

13. Deng Y, Liu W, Liu K, Fang Y-Y, Shang J, Wang K, et al. Clinical characteristics of fatal and recovered cases of coronavirus disease 2019 (COVID-19) in Wuhan, China: a retrospective study. *Chin Med J (Engl).* (2020) <https://doi.org/10.1097/CM9.0000000000000824>.

14. Ding Q, Lu P, Fan Y, Xia Y, Liu M. The clinical characteristics of pneumonia patients co‐infected with 2019 novel coronavirus and influenza virus in Wuhan, China. *J Med Virol.* (2020) <https://doi.org/10.1002/jmv.25781>.

15. Du R, Liang L, Yang C, Li M, Guo G, van Halm-Lutterodt N, et al. Patient Predisposition at Hospital Admission Indirectly Dictates Disease Severity, Clinical Course and Outcomes of COVID-19 Pneumonia Patients in Wuhan, China. *SSRN.* (2020) <https://ssrn.com/abstract=3543584>.

16. Du W, Yu J, Wang H, Zhang X, Zhang S, Li Q, et al. Clinical characteristics of COVID-19 in children compared with adults in Shandong Province, China. *Infection.* (2020) <https://doi.org/10.1007/s15010-020-01427-2>.

17. Fan H, Zhang L, Huang B, Zhu M, Zhou Y, Zhang H, et al. Retrospective Analysis of Clinical Features in 101 Death Cases with COVID-19. *medRxiv.* (2020) <https://doi.org/10.1101/2020.03.09.20033068>.

18. Feng Z, Yu Q, Yao S, Luo L, Duan J, Yan Z, et al. Early prediction of disease progression in 2019 novel coronavirus pneumonia patients outside Wuhan with CT and clinical characteristics. *medRxiv.* (2020) <https://doi.org/10.1101/2020.02.19.20025296>.

19. Fu H, Xu H, Zhang N, Xu H, Li Z, Chen H, et al. Association between Clinical, Laboratory and CT Characteristics and RT-PCR Results in the Follow-up of COVID-19 patients. *medRxiv.* (2020) <https://doi.org/10.1101/2020.03.19.20038315>.

20. Guan W-j, Ni Z-y, Hu Y, Liang W-h, Ou C-q, He J-x, et al. Clinical characteristics of coronavirus disease 2019 in China. *N Engl J Med.* (2020) <https://doi.org/10.1056/NEJMoa2002032>.

21. Guan W-j, Liang W-h, Zhao Y, Liang H-r, Chen Z-s, Li Y-m, et al. Comorbidity and its impact on 1590 patients with Covid-19 in China: A Nationwide Analysis. *Eur Respir J.* (2020) <https://doi.org/10.1183/13993003.00547-2020>.

22. Han R, Huang L, Jiang H, Dong J, Peng H, Zhang D. Early clinical and CT manifestations of coronavirus disease 2019 (COVID-19) pneumonia. *Am J Roentgenol.* (2020) <https://doi.org/10.2214/AJR.20.22961>.

23. Han X, Cao Y, Jiang N, Chen Y, Alwalid O, Zhang X, et al. Novel Coronavirus Pneumonia (COVID-19) Progression Course in 17 Discharged Patients: Comparison of Clinical and Thin-Section CT Features During Recovery. *Clin Infect Dis.* (2020) <https://doi.org/10.1093/cid/ciaa271>.

24. He W, Chen L, Chen L, Yuan G, Fang Y, Chen W, et al. Clinical Characteristics and Outcome of 31 Cases with Covid-19 Diagnosed in Haematology Units in Wuhan, China: A Retrospective Cohort Study. *SSRN.* (2020) <https://ssrn.com/abstract=3558004>.

25. Hu L, Chen S, Fu Y, Gao Z, Long H, Ren H-w, et al. Risk factors associated with clinical outcomes in 323 COVID-19 patients in Wuhan, China. *medRxiv.* (2020) <https://doi.org/10.1101/2020.03.25.20037721>.

26. Huang C, Wang Y, Li X, Ren L, Zhao J, Hu Y, et al. Clinical features of patients infected with 2019 novel coronavirus in Wuhan, China. *Lancet.* (2020) 395:497-506.

27. Huang R, Zhu L, Xue L, Liu L, Yan X, Wang J, et al. Clinical Findings of Patients with Coronavirus Disease 2019 in Jiangsu Province, China: A Retrospective, Multi-Center Study. *SSRN.* (2020) <https://ssrn.com/abstract=3548785>.

28. Huang Y, Tu M, Wang S, Chen S, Zhou W, Chen D, et al. Clinical characteristics of laboratory confirmed positive cases of SARS-CoV-2 infection in Wuhan, China: a retrospective single center analysis. *Travel Med Infect Dis.* (2020) <https://doi.org/10.1016/j.tmaid.2020.101606>.

29. Jin X, Lian J-S, Hu J-H, Gao J, Zheng L, Zhang Y-M, et al. Epidemiological, clinical and virological characteristics of 74 cases of coronavirus-infected disease 2019 (COVID-19) with gastrointestinal symptoms. *Gut.* (2020) <https://doi.org/10.1136/gutjnl-2020-320926>.

30. Chen L, Liu H, Liu W, Liu J, Liu K, Shang J. Analysis of clinical characteristics of 29 cases of new coronavirus pneumonia in 2019. *Zhonghua jie he he hu xi za zhi = Zhonghua jiehe he huxi zazhi = Chinese journal of tuberculosis and respiratory diseases.* (2020) <https://doi.org/10.3760/cma.j.issn.1001-0939.2020.0005>.

31. Li J, Long X, Luo H, Fang F, Lv X, Zhang D, et al. Clinical Characteristics of Deceased Patients Infected with SARS-CoV-2 in Wuhan, China. *SSRN.* (2020) <https://ssrn.com/abstract=3546043>.

32. Li J, Zhang Y, Wang F, Liu B, Li H, Tang G, et al. Sex differences in clinical findings among patients with coronavirus disease 2019 (COVID-19) and severe condition. *medRxiv.* (2020) <https://doi.org/10.1101/2020.02.27.20027524>.

33. Li K, Wu J, Wu F, Guo D, Chen L, Fang Z, et al. The Clinical and Chest CT Features Associated with Severe and Critical COVID-19 Pneumonia. *Investig Radiol.* (2020) <https://doi.org/10.1097/RLI.0000000000000672>.

34. Li Y, Wang J, Wang C, Yang Q, Xu Y, Xu J, et al. Characteristics of Respiratory Virus Infection During the Outbreak of 2019 Novel Coronavirus in Beijing. *Int J Infect Dis.* (2020) <https://doi.org/10.1016/j.ijid.2020.05.008>.

35. Li K, Fang Y, Li W, Pan C, Qin P, Zhong Y, et al. CT image visual quantitative evaluation and clinical classification of coronavirus disease (COVID-19). *Eur Radiol.* (2020) <https://doi.org/10.1007/s00330-020-6817-6>.

36. Lian J, Jin X, Hao S, Cai H, Zhang S, Zheng L, et al. Analysis of Epidemiological and Clinical features in older patients with Corona Virus Disease 2019 (COVID-19) out of Wuhan. *Clin Infect Dis.* (2020) <https://doi.org/10.1093/cid/ciaa242/5811557>.

37. Liang Y, Liang J, Zhou Q, Li X, Lin F, Deng Z, et al. Prevalence and clinical features of 2019 novel coronavirus disease (COVID-19) in the Fever Clinic of a teaching hospital in Beijing: a single-center, retrospective study. *medRxiv.* (2020) <https://doi.org/10.1101/2020.02.25.20027763>.

38. Zhang J, Guo L, Liu H, Lv J, Xu L. Epidemiological and clinical characteristics of COVID-19 in adolescents and young adults. *Cell.* (2020) <https://doi.org/10.1016/j.xinn.2020.04.001>.

39. Liu K, Chen Y, Wu D, Lin R, Wang Z, Pan L. Effects of progressive muscle relaxation on anxiety and sleep quality in patients with COVID-19. *Complement Ther Clin Pract.* (2020) <https://doi.org/10.1016/j.ctcp.2020.101132>.

40. Jian-ya G. Clinical characteristics of 51 patients discharged from hospital with COVID-19 in Chongqing, China. *medRxiv.* (2020) <https://doi.org/10.1101/2020.02.20.20025536>.

41. Li J, Song H, Hu Z. Clinical features and outcomes of 2019 novel coronavirus-infected patients with cardiac injury. *medRxiv.* (2020) <https://doi.org/10.1101/2020.03.11.20030957>.

42. Liu F, Xu A, Zhang Y, Xuan W, Yan T, Pan K, et al. Patients of COVID-19 may benefit from sustained lopinavir-combined regimen and the increase of eosinophil may predict the outcome of COVID-19 progression. *Int J Infect Dis.* (2020) <https://doi.org/10.1016/j.ijid.2020.03.013>.

43. Liu J, Li S, Liu J, Liang B, Wang X, Wang H, et al. Longitudinal characteristics of lymphocyte responses and cytokine profiles in the peripheral blood of SARS-CoV-2 infected patients. *EBioMedicine.* (2020) 102763.

44. Liu J, Ouyang L, Guo P, sheng Wu H, Fu P, liang Chen Y, et al. Epidemiological, Clinical Characteristics and Outcome of Medical Staff Infected with COVID-19 in Wuhan, China: A Retrospective Case Series Analysis. *medRxiv.* (2020) <https://doi.org/10.1101/2020.03.09.20033118>.

45. Liu K, Fang Y-Y, Deng Y, Liu W, Wang M-F, Ma J-P, et al. Clinical characteristics of novel coronavirus cases in tertiary hospitals in Hubei Province. *Chin Med J (Engl).* (2020) <https://doi.org/10.1097/CM9.0000000000000744>.

46. Chen M, Fan Y, Wu X, Zhang L, Guo T, Deng K, et al. Clinical characteristics and risk factors for fatal outcome in patients with 2019-coronavirus infected disease (COVID-19) in Wuhan, China. *SSRN.* (2020) <https://ssrn.com/abstract=3546069>.

47. Lu H, Ai J, Shen Y, Li Y, Li T, Zhou X, et al. A descriptive study of the impact of diseases control and prevention on the epidemics dynamics and clinical features of SARS-CoV-2 outbreak in Shanghai, lessons learned for metropolis epidemics prevention. *medRxiv.* (2020) <https://doi.org/10.1101/2020.02.19.20025031>.

48. Lu C, Yang W, Hu Y, Hui J, Zhou G, Shu J, et al. Coronavirus Disease 2019 (COVID-19) Pneumonia: Early Stage Chest CT Imaging Features and Clinical Relevance. *SSRN.* (2020) <https://ssrn.com/abstract=3543606>.

49. Mao B, Liu Y, Chai Y-h, Jin X-y, Luo HW, Yang J-w, et al. Early Discern COVID-19 from the Suspected Patients via Fever Clinics: A Multicenter Cohort Study from Shanghai. *SSRN.* (2020) <https://ssrn.com/abstract=3550037>.

50. Miao C, Zhuang J, Jin M, Xiong H, Huang P, Zhao Q, et al. A comparative multi-centre study on the clinical and imaging features of comfirmed and uncomfirmed patients with COVID-19. *medRxiv.* (2020).

51. Liu M, He P, Liu H, Wang X, Li F, Chen S, et al. Clinical characteristics of 30 medical workers infected with new coronavirus pneumonia. *Zhonghua jie he he hu xi za zhi = Zhonghua jiehe he huxi zazhi = Chinese journal of tuberculosis and respiratory diseases.* (2020) 43:1-7.

52. Mo P, Xing Y, Xiao Y, Deng L, Zhao Q, Wang H, et al. Clinical characteristics of refractory COVID-19 pneumonia in Wuhan, China. *Clin Infect Dis.* (2020) <https://doi.org/10.1093/cid/ciaa270/5805508>.

53. Qian G-Q, Yang N-B, Ding F, Ma AHY, Wang Z-Y, Shen Y-F, et al. Epidemiologic and Clinical Characteristics of 91 Hospitalized Patients with COVID-19 in Zhejiang, China: A retrospective, multi-centre case series. *QJM.* (2020).

54. Qin C, Zhou L, Hu Z, Zhang S, Yang S, Tao Y, et al. Dysregulation of immune response in patients with COVID-19 in Wuhan, China. *Clin Infect Dis.* (2020) <https://doi.org/10.1093/cid/ciaa248/5803306>.

55. Qin X, Qiu S, Yuan Y, Zong Y, Tuo Z, Li J, et al. Clinical characteristics and treatment of patients infected with COVID-19 in Shishou, China. *SSRN.* (2020) <https://ssrn.com/abstract=3541147>.

56. Shi H, Han X, Jiang N, Cao Y, Alwalid O, Gu J, et al. Radiological findings from 81 patients with COVID-19 pneumonia in Wuhan, China: a descriptive study. *Lancet Infect Dis.* (2020) <https://doi.org/10.1016/S473-3099(20)30086-4>.

57. Shi Q, Zhao K, Yu J, Feng J, Zhao K, Zhang X, et al. Clinical characteristics of 101 non-surviving hospitalized patients with COVID-19: A single center, retrospective study. *medRxiv.* (2020) <https://doi.org/10.1101/2020.03.04.20031039>.

58. Shu L, Wang X, Li M, Chen X, Shi L, Wu M, et al. Clinical Characteristics of 545 Cases Confirmed COVID-19 in Wuhan Stadium Cabin Hospital. *SSRN.* (2020) <https://ssrn.com/abstract=3552844>.

59. Song F, Shi N, Shan F, Zhang Z, Shen J, Lu H, et al. Emerging 2019 novel coronavirus (2019-nCoV) pneumonia. *Radiology.* (2020) 295:210-7.

60. Tian S, Chang Z, Wang Y, Wu M, Zhang W, Zhou G, et al. Clinical characteristics and reasons of different duration from onset to release from quarantine for patients with COVID-19 Outside Hubei province, China. *medRxiv.* (2020) <https://doi.org/10.1101/2020.03.21.20038778>.

61. Wan S, Xiang Y, Fang W, Zheng Y, Li B, Hu Y, et al. Clinical Features and Treatment of COVID‐19 Patients in Northeast Chongqing. *J Med Virol.* (2020) <https://doi.org/10.1002/jmv.25783>.

62. Wang D, Hu B, Hu C, Zhu F, Liu X, Zhang J, et al. Clinical characteristics of 138 hospitalized patients with 2019 novel coronavirus–infected pneumonia in Wuhan, China. *JAMA.* (2020) 323:1061-9.

63. Wang W, He J, Wu S. The definition and risks of cytokine release syndrome-like in 11 COVID-19-infected pneumonia critically ill patients: disease characteristics and retrospective analysis. *medRxiv.* (2020) <https://doi.org/10.1101/2020.02.26.20026989>.

64. Wang Y, Dong C, Hu Y, Li C, Ren Q, Zhang X, et al. Temporal changes of CT findings in 90 patients with COVID-19 pneumonia: a longitudinal study. *Radiology.* (2020) <https://doi.org/10.1148/radiol.2020200843>.

65. Wang Z, Yang B, Li Q, Wen L, Zhang R. Clinical features of 69 cases with coronavirus disease 2019 in Wuhan, China. *Clin Infect Dis.* (2020) <https://doi.org/10.1093/cid/ciaa272/5807944>.

66. Wang L, He W, Yu X, Hu D, Bao M, Liu H, et al. Coronavirus Disease 2019 in elderly patients: characteristics and prognostic factors based on 4-week follow-up. *J Infect.* (2020) <https://doi.org/10.1016/j.jinf.2020.03.019>.

67. Wei L, Jin D, Zhang J, Wang B, Sun M, Li X, et al. Clinical Findings of 100 Mild Cases of COVID-19 in Wuhan: A Descriptive Study. *SSRN.* (2020) <https://ssrn.com/abstract=3551332>.

68. Wei X-S, Wang X, Niu Y-R, Ye L-L, Peng W-B, Wang Z-H, et al. Clinical Characteristics of SARS-CoV-2 Infected Pneumonia with Diarrhea. *SSRN.* (2020) <https://ssrn.com/abstract=3546120>.

69. Xu H, Huang S, Liu S, Deng J, Jiao B, Ai L, et al. Evaluation of the clinical characteristics of suspected or confirmed cases of COVID-19 during home care with isolation: A new retrospective analysis based on O2O. *medRxiv.* (2020) <https://doi.org/10.1101/2020.02.26.20028084>.

70. Xu Y, Li Y-r, Zeng Q, Lu Z-b, Li Y-z, Wu W, et al. Clinical characteristics of SARS-CoV-2 pneumonia compared to controls in Chinese Han population. *medRxiv.* (2020) <https://doi.org/10.1101/2020.03.08.20031658>.

71. Yang X, Yu Y, Xu J, Shu H, Liu H, Wu Y, et al. Clinical course and outcomes of critically ill patients with SARS-CoV-2 pneumonia in Wuhan, China: a single-centered, retrospective, observational study. *Lancet Respir Med.* (2020) <https://doi.org/10.1016/S2213-600(20)30079-5>.

72. Yang P, Ding Y, Xu Z, Pu R, Li P, Yan J, et al. Epidemiological and clinical features of COVID-19 patients with and without pneumonia in Beijing, China. *medRxiv.* (2020) <https://doi.org/10.1101/2020.02.28.20028068>.

73. Yao T, Gao Y, Cui Q, Shen J, Peng B, Chen Y, et al. Clinical Characteristics of 55 Cases of Deaths with COVID-19 Pneumonia in Wuhan, China: Retrospective Case Series. *SSRN.* (2020) <https://ssrn.com/abstract=3550019>.

74. Yu F, Yan L, Wang N, Yang S, Wang L, Tang Y, et al. Quantitative detection and viral load analysis of SARS-CoV-2 in infected patients. *Clin Infect Dis.* (2020) <https://doi.org/10.1093/cid/ciaa345/5812997>.

75. Li Y, Wang W, Lei Y, Zhang B, Yang J, Hu J, et al. Comparison of the clinical characteristics between RNA positive and negative patients clinically diagnosed with 2019 novel coronavirus pneumonia. *Zhonghua jie he he hu xi za zhi = Zhonghua jiehe he huxi zazhi = Chinese journal of tuberculosis and respiratory diseases.* (2020) 43:1-10.

76. Zhang F, He L, Ouyang Y, Gong J, Li X, Wei Y, et al. Clinical Features of 81 Hospitalized Patients with 2019 Novel Coronavirus-Infected Pneumonia in Jingzhou, China: A Descriptive Study. *SSRN.* (2020) <https://ssrn.com/abstract=3544834>.

77. Zhang G, Hu C, Luo L, Fang F, Chen Y, Li J, et al. Clinical features and outcomes of 221 patients with COVID-19 in Wuhan, China. *medRxiv.* (2020) <https://doi.org/10.1101/2020.03.02.20030452>.

78. Zhang X, Cai H, Hu J, Lian J, Gu J, Zhang S, et al. Epidemiological, clinical characteristics of cases of SARS-CoV-2 infection with abnormal imaging findings. *Int J Infect Dis.* (2020) <https://doi.org/10.1016/j.ijid.2020.03.040>.

79. Zhang Y. Gastrointestinal tract symptoms in coronavirus disease 2019: Analysis of clinical symptoms in adult patients. *medRxiv.* (2020) <https://doi.org/10.1101/2020.03.23.20040279>.

80. Zhang L, Sun W, Chen L, Wang Q, Liu Y, Zhao S, et al. Clinical Features and a Simple Model for Predicting the Mortality of Coronavirus Disease 2019 Patients on Admission. *SSRN.* (2020) <https://ssrn.com/abstract=3551386>.

81. Zhao D, Yao F, Wang L, Zheng L, Gao Y, Ye J, et al. A comparative study on the clinical features of COVID-19 pneumonia to other pneumonias. *Clin Infect Dis.* (2020) <https://doi.org/10.1093/cid/ciaa247/5803302>.

82. Zhao W, He L, Xie X, Liu J. The Viral Load of 2019 Novel Coronavirus (COVID-19) has the potential to predict the clinical outcomes. *SSRN.* (2020) <https://ssrn.com/abstract=3546047>.

83. Zhao H, Lu J, Hongjun Z, Lu X, Li T, Rao B, et al. A New Features of SARS-CoV-2 Infection in Wenzhou, China. *Research Square.* (2020) <https://doi.org/10.21203/rs.2.3945/v1>.

84. Zhong Q, Liu YY, Luo Q, Zou YF, Jiang HX, Li H, et al. Spinal anaesthesia for patients with coronavirus disease 2019 and possible transmission rates in anaesthetists: retrospective, single-centre, observational cohort study. *Br J Anaesth.* (2020) <https://doi.org/10.1016/j.bja.2020.03.007>.

85. Zhou Z, Zhao N, Shu Y, Han S, Chen B, Shu X. Effect of gastrointestinal symptoms on patients infected with COVID-19. *Gastroenterology.* (2020) <https://doi.org/10.1053/j.gastro.2020.03.020>.

86. Zhu W, Xie K, Lu H, Xu L, Zhou S, Fang S. Initial clinical features of suspected Coronavirus Disease 2019 in two emergency departments outside of Hubei, China. *J Med Virol.* (2020) <https://doi.org/10.1002/jmv.25763>.
