# Supplementary material for: Prevalence of Headache in Patients With Coronavirus Disease 2019 (COVID-19): A Systematic Review and Meta-Analysis of 14,275 Patients
Source: Front Neurol. 2020 Nov 27;11:562634. doi: 10.3389/fneur.2020.562634 (PMC7728918; doi:10.3389/fneur.2020.562634)
Supplement: Supplementary file 3 [file Table_3.DOCX]

| **Supplementary Table 3. Quality assessment of the included cross-sectional studies** | | | | | | | | | | |
| --- | --- | --- | --- | --- | --- | --- | --- | --- | --- | --- |
| **No.** | **Study ID** | **Questions assessing included cross-sectional studies** | | | | | | | | **Yes (%)** |
|  |  | **1** | **2** | **3** | **4** | **5** | **6** | **7** | **8** |  |
| 1 | Bhatraju 2020 | Y | Y | Y | Y | N | N | N | Y | 62·5 |
| 2 | Cao 2020 | Y | Y | Y | Y | N | N | Y | Y | 75·0 |
| 3 | Chen 2020 | Y | Y | Y | Y | N | N | Y | Y | 75·0 |
| 4 | Chen 2020a | Y | Y | Y | Y | Y | Y | Y | Y | 100·0 |
| 5 | Chen 2020b | Y | Y | Y | Y | N | N | Y | Y | 75·0 |
| 6 | Chen 2020c | Y | Y | Y | Y | N | N | N | Y | 62·5 |
| 7 | Chen 2020d | Y | Y | Y | N | N | N | Y | Y | 62·5 |
| 8 | Chen 2020e | Y | Y | Y | Y | N | N | Y | Y | 75·0 |
| 9 | Chen 2020f | Y | Y | Y | Y | N | N | Y | Y | 75·0 |
| 10 | Chen 2020g | Y | Y | Y | Y | N | N | N | Y | 62·5 |
| 11 | Deng 2020 | Y | Y | Y | Y | N | N | N | Y | 62·5 |
| 12 | Du 2020 | Y | Y | Y | Y | Y | Y | Y | Y | 100·0 |
| 13 | Du 2020a | Y | Y | Y | Y | N | N | Y | Y | 75·0 |
| 14 | Fan 2020 | Y | Y | Y | Y | N | N | Y | Y | 75·0 |
| 15 | Feng 2020 | Y | Y | Y | Y | Y | N | N | Y | 75·0 |
| 16 | Fu 2020 | Y | Y | Y | Y | N | N | N | Y | 62·5 |
| 17 | Guan 2020 | Y | Y | Y | Y | N | N | Y | Y | 75·0 |
| 18 | Han 2020 | Y | Y | Y | Y | N | N | Y | N | 62·5 |
| 19 | Hu 2020 | Y | Y | Y | Y | Y | Y | Y | Y | 100·0 |
| 20 | Huang 2020 | Y | Y | Y | Y | N | N | Y | Y | 75·0 |
| 21 | Huang 2020a | Y | Y | Y | Y | Y | N | N | Y | 75·0 |
| 22 | Huang 2020b | Y | Y | N | Y | N | N | Y | N | 50·0 |
| 23 | Jin 2020 | Y | Y | Y | Y | Y | Y | Y | Y | 100·0 |
| 24 | Lei 2020 | Y | Y | Y | Y | N | N | N | Y | 62·5 |
| 25 | Li 2020 | Y | Y | Y | Y | N | N | Y | Y | 75·0 |
| 26 | Li 2020a | Y | Y | Y | Y | Y | N | N | Y | 75·0 |
| 27 | Li 2020b | Y | Y | Y | Y | Y | Y | N | Y | 87·5 |
| 28 | Li 2020c | Y | Y | Y | Y | N | N | Y | Y | 75·0 |
| 29 | Li 2020d | Y | Y | Y | Y | N | N | Y | Y | 75·0 |
| 30 | Lian 2020 | Y | Y | Y | Y | N | N | Y | Y | 75·0 |
| 31 | Liang 2020 | Y | Y | Y | Y | N | N | Y | Y | 75·0 |
| 32 | Liao 2020 | Y | Y | Y | Y | N | N | N | Y | 62·5 |
| 33 | Liu 2020a | Y | Y | Y | Y | N | N | N | Y | 62·5 |
| 34 | Liu 2020b | Y | Y | Y | Y | N | N | Y | Y | 75·0 |
| 35 | Liu 2020d | Y | Y | Y | Y | Y | Y | Y | Y | 100·0 |
| 36 | Liu 2020e | Y | Y | Y | Y | Y | Y | N | Y | 87·5 |
| 37 | Liu 2020f | Y | Y | Y | Y | N | N | Y | Y | 75·0 |
| 38 | Lu 2020 | Y | Y | Y | Y | Y | Y | Y | Y | 100·0 |
| 39 | Lu 2020b | Y | Y | N | Y | N | N | N | Y | 50·0 |
| 40 | Miao 2020 | Y | Y | N | Y | N | N | Y | Y | 62·5 |
| 41 | Min 2020 | Y | Y | N | Y | N | N | Y | Y | 62·5 |
| 42 | Mo 2020 | Y | Y | N | Y | Y | Y | Y | Y | 87·5 |
| 43 | Qian 2020 | Y | Y | Y | Y | N | N | Y | Y | 75·0 |
| 44 | Qin 2020 | Y | Y | Y | Y | N | N | N | Y | 62·5 |
| 45 | Qin 2020a | Y | Y | Y | Y | N | N | N | Y | 62·5 |
| 46 | Shi 2020 | Y | Y | Y | Y | N | N | Y | Y | 75·0 |
| 47 | Shi 2020a | Y | Y | Y | N | Y | Y | Y | Y | 87·5 |
| 48 | Shu 2020 | Y | Y | Y | Y | N | N | N | Y | 62·5 |
| 49 | Song 2020 | Y | Y | N | Y | N | N | Y | Y | 62·5 |
| 50 | Tian 2020 | Y | Y | Y | Y | N | N | Y | Y | 75·0 |
| 51 | Wan 2020 | Y | Y | Y | Y | N | N | Y | Y | 75·0 |
| 52 | Wang 2020 | Y | Y | Y | Y | U | N | Y | Y | 75·0 |
| 53 | Wang 2020b | Y | Y | Y | Y | N | N | N | Y | 62·5 |
| 54 | Wang 2020c | Y | Y | Y | Y | N | N | Y | Y | 75·0 |
| 55 | Wang 2020d | Y | Y | Y | Y | Y | Y | N | Y | 87·5 |
| 56 | Wei 2020 | Y | Y | Y | Y | N | N | N | Y | 62·5 |
| 57 | Wei 2020a | Y | Y | Y | Y | N | N | N | Y | 62·5 |
| 58 | Xu 2020 | Y | Y | Y | Y | N | N | Y | Y | 75·0 |
| 59 | Xu 2020a | Y | Y | Y | Y | U | N | N | Y | 62·5 |
| 60 | Yang 2020 | Y | Y | Y | N | N | N | N | Y | 50·0 |
| 61 | Yang 2020a | Y | Y | Y | Y | Y | Y | Y | Y | 100·0 |
| 62 | Yao 2020 | Y | Y | Y | Y | N | N | N | Y | 62·5 |
| 63 | Yu 2020 | Y | Y | N | Y | N | N | N | N | 37·5 |
| 64 | Yuanyuan 2020 | Y | Y | Y | Y | N | N | Y | Y | 75·0 |
| 65 | Zhang 2020 | Y | Y | Y | Y | N | N | N | Y | 62·5 |
| 66 | Zhang 2020a | Y | Y | Y | Y | N | N | Y | Y | 75·0 |
| 67 | Zhang 2020b | Y | Y | Y | Y | Y | Y | N | Y | 87·5 |
| 68 | Zhang 2020c | Y | Y | Y | Y | Y | Y | N | Y | 87·5 |
| 69 | Zhang 2020d | Y | Y | Y | Y | Y | Y | N | Y | 87·5 |
| 70 | Zhao 2020 | Y | Y | Y | Y | N | N | N | Y | 62·5 |
| 71 | Zhao 2020a | Y | Y | Y | Y | N | N | N | Y | 62·5 |
| 72 | Zhao 2020b | Y | Y | Y | Y | N | N | N | Y | 62·5 |
| 73 | Zhou 2020 | Y | Y | Y | Y | N | N | N | Y | 62·5 |
| 74 | Zhu 2020 | Y | Y | Y | Y | N | N | Y | Y | 75·0 |
| 1. Were the criteria for inclusion in the sample clearly defined? 2. Were the study subjects and the setting described in detail? 3. Was the exposure measured in a valid and reliable way? 4. Were objective, standard criteria used for measurement of the condition? 5. Were confounding factors identified? 6. Were strategies to deal with confounding factors stated? 7. Were the outcomes measured in a valid and reliable way? 8. Was appropriate statistical analysis used? Y=Yes; N=No; U=Unclear. | | | | | | | | | | |
