# Supplementary material for: Prevalence of Headache in Patients With Coronavirus Disease 2019 (COVID-19): A Systematic Review and Meta-Analysis of 14,275 Patients
Source: Front Neurol. 2020 Nov 27;11:562634. doi: 10.3389/fneur.2020.562634 (PMC7728918; doi:10.3389/fneur.2020.562634)
Supplement: Supplementary file 4 [file Table_4.DOCX]

| **Supplementary Table 4. Quality assessment of the included cohort studies** | | | | | | | | | | | | | |
| --- | --- | --- | --- | --- | --- | --- | --- | --- | --- | --- | --- | --- | --- |
| **No.** | **Study ID** | **Questions assessing included cohort studies** | | | | | | | | | | | **Yes (%)** |
|  |  | **1** | **2** | **3** | **4** | **5** | **6** | **7** | **8** | **9** | **10** | **11** |  |
| 1 | Guan 2020a | Y | Y | Y | Y | Y | Y | Y | N | Y | NA | Y | 90·0 |
| 2 | He 2020 | Y | Y | Y | Y | Y | Y | Y | Y | Y | N | Y | 90·0 |
| 3 | Lu 2020a | Y | Y | Y | U | N | Y | Y | Y | Y | NA | Y | 80·0 |
| 4 | Mao 2020 | Y | Y | Y | Y | Y | Y | Y | Y | Y | NA | Y | 100·0 |
| 5 | Zhong 2020 | Y | Y | Y | N | N | Y | Y | N | Y | NA | Y | 70·0 |
| 1. Were the two groups similar and recruited from the same population? 2. Were the exposures measured similarly to assign people to both exposed and unexposed groups? 3. Was the exposure measured in a valid and reliable way? 4. Were confounding factors identified? 5. Were strategies to deal with confounding factors stated? 6. Were the groups/participants free of the outcome at the start of the study (or at the moment of exposure)? 7. Were the outcomes measured in a valid and reliable way? 8. Was the follow up time reported and sufficient to be long enough for outcomes to occur? 9. Was follow up complete, and if not, were the reasons to loss to follow up described and explored? 10. Were strategies to address incomplete follow up utilized? 11. Was appropriate statistical analysis used? Y=Yes; N=No; U=Unclear; NA: Not applicable. | | | | | | | | | | | | | |
