# Supplementary material for: Prevalence of Headache in Patients With Coronavirus Disease 2019 (COVID-19): A Systematic Review and Meta-Analysis of 14,275 Patients
Source: Front Neurol. 2020 Nov 27;11:562634. doi: 10.3389/fneur.2020.562634 (PMC7728918; doi:10.3389/fneur.2020.562634)
Supplement: Supplementary file 5 [file Table_5.DOCX]

| **Supplementary Table 5. Quality assessment of the included case series** | | | | | | | | | | | | |
| --- | --- | --- | --- | --- | --- | --- | --- | --- | --- | --- | --- | --- |
| **No.** | **Study ID** | **Questions assessing included case series** | | | | | | | | | | **Yes (%)** |
|  |  | **1** | **2** | **3** | **4** | **5** | **6** | **7** | **8** | **9** | **10** |  |
| 1 | Chung 2020 | Y | Y | N | Y | Y | Y | Y | Y | Y | N | 80·0 |
| 2 | Ding 2020 | Y | Y | Y | N | N | Y | Y | Y | Y | Y | 80·0 |
| 3 | Han 2020a | Y | Y | Y | Y | Y | Y | Y | Y | N | Y | 90·0 |
| 4 | Liu 2020c | Y | Y | N | Y | Y | Y | Y | Y | Y | Y | 90·0 |
| 5 | Wang 2020a | Y | Y | Y | Y | Y | Y | Y | Y | Y | Y | 100·0 |
| 1. Were there clear criteria for inclusion in the case series? 2. Was the condition measured in a standard, reliable way for all participants included in the case series? 3. Were valid methods used for identification of the condition for all participants included in the case series? 4. Did the case series have consecutive inclusion of participants? 5. Did the case series have complete inclusion of participants? 6. Was there clear reporting of the demographics of the participants in the study? 7. Was there clear reporting of clinical information of the participants? 8. Were the outcomes or follow up results of cases clearly reported? 9. Was there clear reporting of the presenting site(s)/clinic(s) demographic information? 10. Was statistical analysis appropriate? Y=Yes; N=No; U=Unclear. | | | | | | | | | | | | |
