# Supplementary material for: Prevalence of Headache in Patients With Coronavirus Disease 2019 (COVID-19): A Systematic Review and Meta-Analysis of 14,275 Patients
Source: Front Neurol. 2020 Nov 27;11:562634. doi: 10.3389/fneur.2020.562634 (PMC7728918; doi:10.3389/fneur.2020.562634)
Supplement: Supplementary file 7 [file Table_7.DOCX]

| **Supplementary Table 7. Quality assessment of the included case-control studies** | | | | | | | | | | | | |
| --- | --- | --- | --- | --- | --- | --- | --- | --- | --- | --- | --- | --- |
| **No.** | **Study ID** | **Questions assessing included case-control studies** | | | | | | | | | | **Yes (%)** |
|  |  | **1** | **2** | **3** | **4** | **5** | **6** | **7** | **8** | **9** | **10** |  |
| 1 | Cheng 2020 | Y | Y | Y | Y | Y | U | N | Y | Y | Y | 80·0 |
| 1. Were the groups comparable other than the presence of disease in cases or the absence of disease in controls? 2. Were cases and controls matched appropriately? 3. Were the same criteria used for identification of cases and controls? 4. Was exposure measured in a standard, valid and reliable way? 5. Was exposure measured in the same way for cases and controls? 6. Were confounding factors identified? 7. Were strategies to deal with confounding factors stated? 8. Were outcomes assessed in a standard, valid and reliable way for cases and controls? 9. Was the exposure period of interest long enough to be meaningful? 10. Was appropriate statistical analysis used? Y=Yes; N=No; U=Unclear. | | | | | | | | | | | | |
