# Supplementary figures and images for: Prevalence of Headache in Patients With Coronavirus Disease 2019 (COVID-19): A Systematic Review and Meta-Analysis of 14,275 Patients
Source: Front Neurol. 2020 Nov 27;11:562634. doi: 10.3389/fneur.2020.562634 (PMC7728918; doi:10.3389/fneur.2020.562634)

A

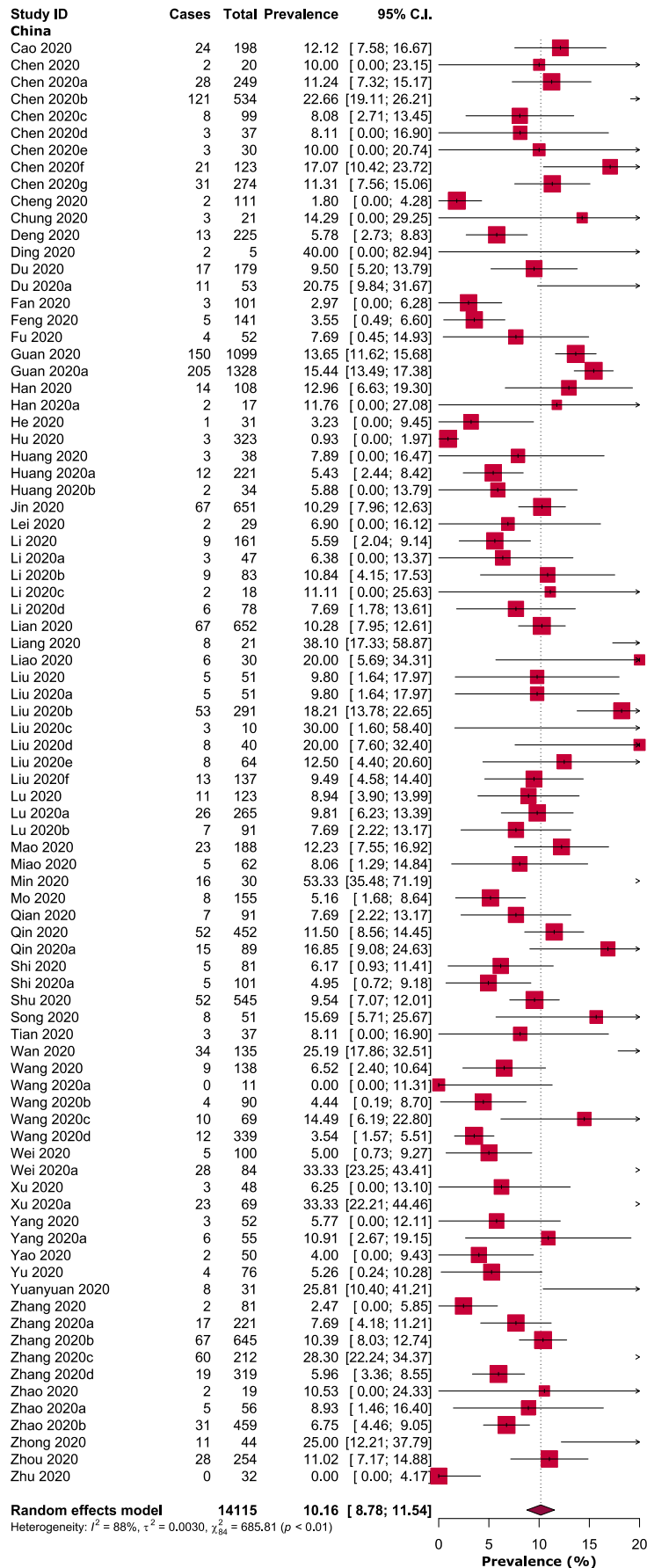

**B**

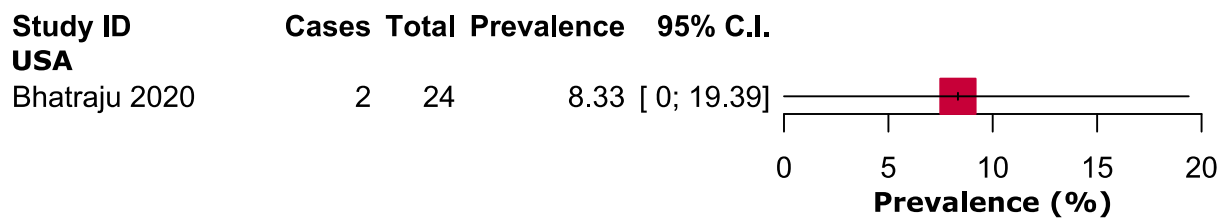

**Supplementary Figure 1.** Prevalence of headache in COVID-19 patients from (A) China and (B) USA.

Supplement: Supplementary file 8 [file Image_1.PDF]
