# Supplementary material for: Prevalence of Headache in Patients With Coronavirus Disease 2019 (COVID-19): A Systematic Review and Meta-Analysis of 14,275 Patients
Source: Front Neurol. 2020 Nov 27;11:562634. doi: 10.3389/fneur.2020.562634 (PMC7728918; doi:10.3389/fneur.2020.562634)
Supplement: Supplementary file 9 [file Image_2.PDF]

A

Study ID	Cases	Total	Prevalence	95% C.I.
**Hubei**				
Chen 2020	2	20	10.00	[0.00; 23.15]
Chen 2020b	121	534	22.66	[19.11; 26.21]
Chen 2020c	8	99	8.08	[2.71; 13.45]
Chen 2020e	3	30	10.00	[0.00; 20.74]
Chen 2020f	21	123	17.07	[10.42; 23.72]
Chen 2020g	31	274	11.31	[7.56; 15.06]
Cheng 2020	2	111	1.80	[0.00; 4.28]
Deng 2020	13	225	5.78	[2.73; 8.83]
Ding 2020	2	5	40.00	[0.00; 82.94]
Du 2020	17	179	9.50	[5.20; 13.79]
Fan 2020	3	101	2.97	[0.00; 6.28]
Han 2020	14	108	12.96	[6.63; 19.30]
Han 2020a	2	17	11.76	[0.00; 27.08]
He 2020	1	31	3.23	[0.00; 9.45]
Hu 2020	3	323	0.93	[0.00; 1.97]
Huang 2020	3	38	7.89	[0.00; 16.47]
Huang 2020b	2	34	5.88	[0.00; 13.79]
Lei 2020	2	29	6.90	[0.00; 16.12]
Li 2020	9	161	5.59	[2.04; 9.14]
Liu 2020d	8	40	20.00	[7.60; 32.40]
Liu 2020e	8	64	12.50	[4.40; 20.60]
Liu 2020f	13	137	9.49	[4.58; 14.40]
Lu 2020	11	123	8.94	[3.90; 13.99]
Lu 2020b	7	91	7.69	[2.22; 13.17]
Min 2020	16	30	53.33	[35.48; 71.19]
Mo 2020	8	155	5.16	[1.68; 8.64]
Qian 2020	7	91	7.69	[2.22; 13.17]
Qin 2020	52	452	11.50	[8.56; 14.45]
Qin 2020a	15	89	16.85	[9.08; 24.63]
Shi 2020	5	81	6.17	[0.93; 11.41]
Shi 2020a	5	101	4.95	[0.72; 9.18]
Shu 2020	52	545	9.54	[7.07; 12.01]
Wang 2020	9	138	6.52	[2.40; 10.64]
Wang 2020b	4	90	4.44	[0.19; 8.70]
Wang 2020c	10	69	14.49	[6.19; 22.80]
Wang 2020d	12	339	3.54	[1.57; 5.51]
Wei 2020	5	100	5.00	[0.73; 9.27]
Wei 2020a	28	84	33.33	[23.25; 43.41]
Xu 2020	3	48	6.25	[0.00; 13.10]
Yang 2020	3	52	5.77	[0.00; 12.11]
Yao 2020	2	55	3.64	[0.00; 8.58]
Yuanyuan 2020	8	31	25.81	[10.40; 41.21]
Zhang 2020	2	81	2.47	[0.00; 5.85]
Zhang 2020a	17	221	7.69	[4.18; 11.21]
Zhang 2020c	60	212	28.30	[22.24; 34.37]
Zhang 2020d	19	319	5.96	[3.36; 8.55]
Zhong 2020	11	44	25.00	[12.21; 37.79]
Zhou 2020	28	254	11.02	[7.17; 14.88]
**Random effects model**		**6578**	**9.56**	**[7.73; 11.39]**

Heterogeneity: $I^2 = 88\%$, $\tau^2 = 0.0031$, $\chi^2_{47} = 405.60$ ($p < 0.01$)

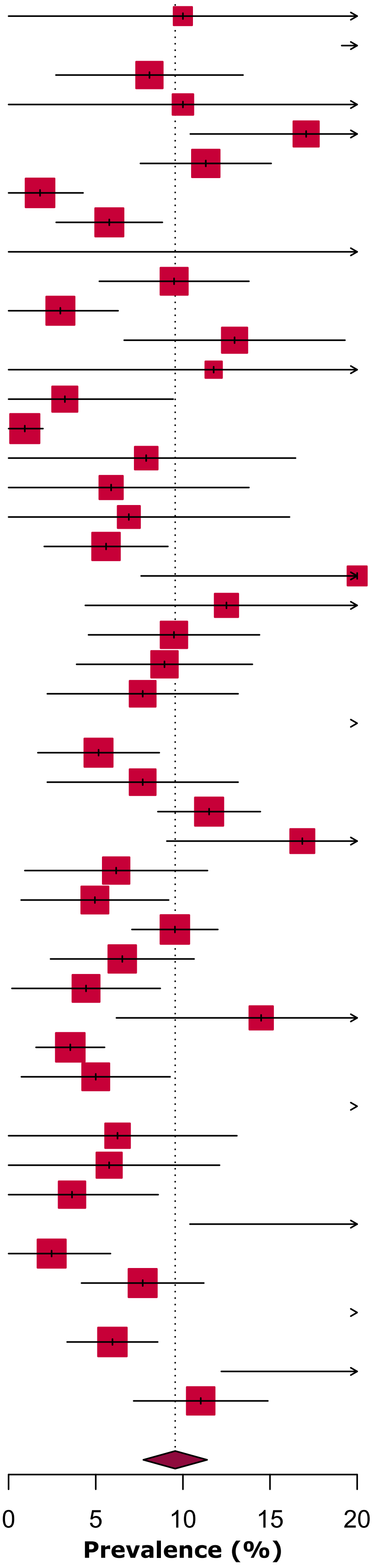

B

Study ID	Cases	Total	Prevalence	95% C.I.
**Shanghai**				
Cao 2020	24	198	12.12	[7.58; 16.67]
Chen 2020a	28	249	11.24	[7.32; 15.17]
Lu 2020a	26	265	9.81	[6.23; 13.39]
Mao 2020	23	188	12.23	[7.55; 16.92]
Miao 2020	5	62	8.06	[1.29; 14.84]
Song 2020	8	51	15.69	[5.71; 25.67]
**Random effects model**		**1013**	**11.06**	**[9.13; 12.99]**

Heterogeneity: $I^2 = 0\%$, $\tau^2 = 0$, $\chi^2_5 = 2.50$ ($p = 0.78$)

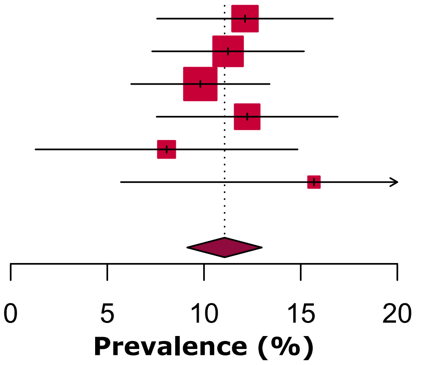

C

Study ID	Cases	Total	Prevalence	95% C.I.
**Zhejiang**				
Jin 2020	67	651	10.29	[7.96; 12.63]
Lian 2020	75	788	9.52	[7.47; 11.57]
Liu 2020c	3	10	30.00	[1.60; 58.40]
Zhang 2020b	67	645	10.39	[8.03; 12.74]
Zhao 2020b	31	459	6.75	[4.46; 9.05]
**Random effects model**		**2553**	**9.31**	**[7.56; 11.07]**

Heterogeneity: $I^2 = 52\%$, $\tau^2 = 0.0002$, $\chi^2_4 = 8.32$ ($p = 0.08$)

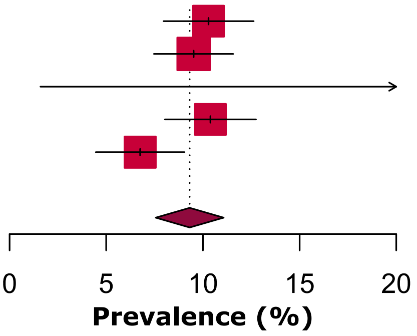

D

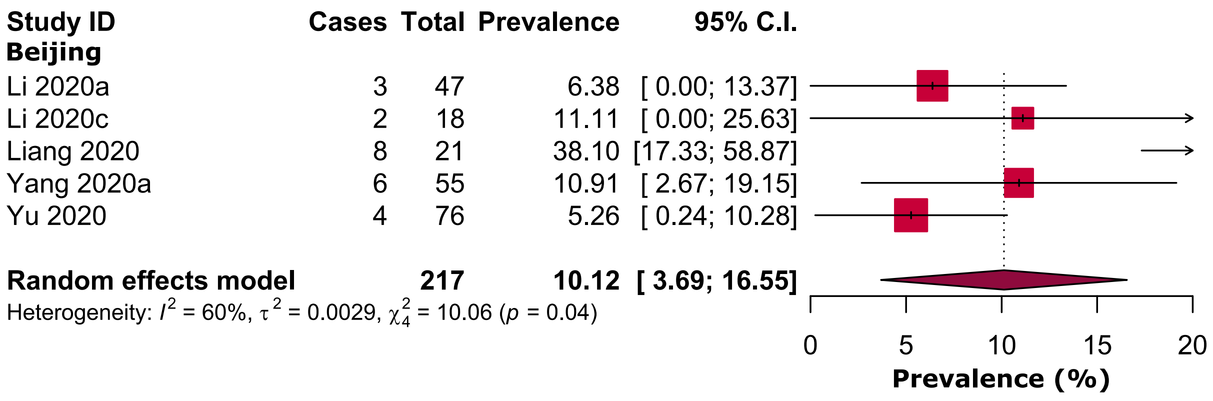
Study ID
Cases
Total
Prevalence
95% C.I.
Beijing
Li 2020a 3 47 6.38 [0.00; 13.37]
Li 2020c 2 18 11.11 [0.00; 25.63]
Liang 2020 8 21 38.10 [17.33; 58.87]
Yang 2020a 6 55 10.91 [2.67; 19.15]
Yu 2020 4 76 5.26 [0.24; 10.28]
Random effects model 217 10.12 [3.69; 16.55]
Heterogeneity: I^2 = 60%, τ^2 = 0.0029, χ^2_4 = 10.06 (p = 0.04)
0
5
10
15
20
Prevalence (%)

E

Study ID	Cases	Total	Prevalence	95% C.I.
**Chongqing**				
Li 2020b	9	83	10.84	[4.15; 17.53]
Liao 2020	6	30	20.00	[5.69; 34.31]
Liu 2020a	5	51	9.80	[1.64; 17.97]
Wan 2020	34	135	25.19	[17.86; 32.51]
**Random effects model**		**299**	**16.11**	**[8.01; 24.21]**

Heterogeneity: $I^2 = 72\%$, $\tau^2 = 0.0047$, $\chi^2_3 = 10.82$ ($p = 0.01$)

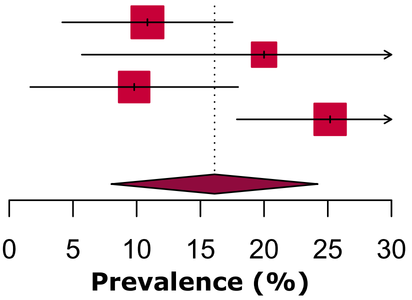

F

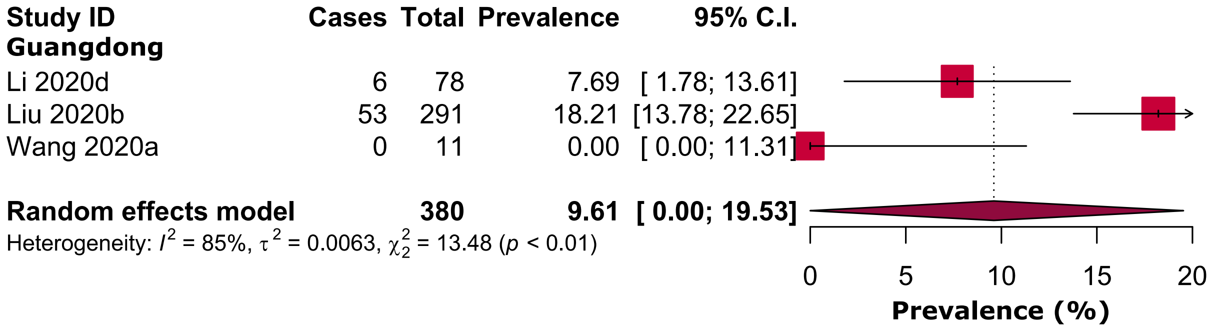
Study ID
Cases Total Prevalence 95% C.I.
Guangdong
Li 2020d 6 78 7.69 [1.78; 13.61]
Liu 2020b 53 291 18.21 [13.78; 22.65]
Wang 2020a 0 11 0.00 [0.00; 11.31]
Random effects model 380 9.61 [0.00; 19.53]
Heterogeneity: I^2 = 85%, τ^2 = 0.0063, χ^2_2 = 13.48 (p < 0.01)
0 5 10 15 20
Prevalence (%)

G

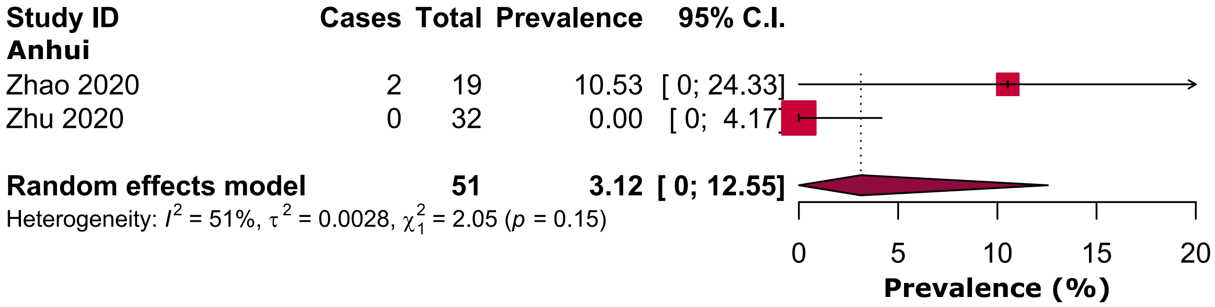
Study ID
Cases
Total
Prevalence
95% C.I.
Anhui
Zhao 2020 2 19 10.53 [0; 24.33]
Zhu 2020 0 32 0.00 [0; 4.17]
Random effects model 51 3.12 [0; 12.55]
Heterogeneity: I^2 = 51%, τ^2 = 0.0028, χ_1^2 = 2.05 (p = 0.15)
0
5
10
15
20
Prevalence (%)

H

Study ID	Cases	Total	Prevalence	95% C.I.
**Hunan**				
Feng 2020	5	141	3.55	[0.49; 6.60]
Zhao 2020a	5	56	8.93	[1.46; 16.40]
**Random effects model**		**197**	**5.11**	**[0.32; 9.91]**

Heterogeneity: $I^2 = 42\%$, $\tau^2 = 0.0006$, $\chi_1^2 = 1.71$ ($p = 0.19$)

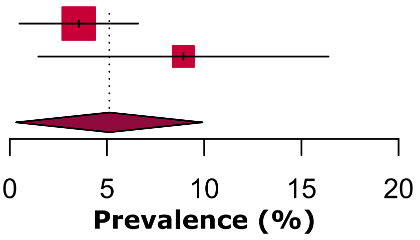

I

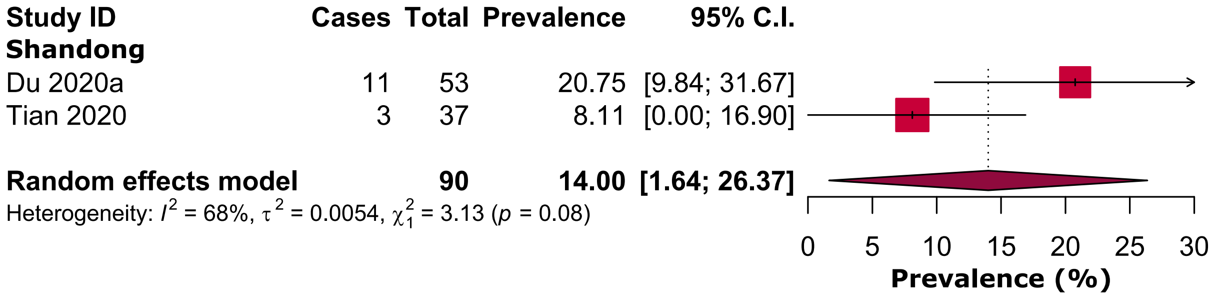
Study ID Cases Total Prevalence 95% C.I.
Shandong
Du 2020a 11 53 20.75 [9.84; 31.67]
Tian 2020 3 37 8.11 [0.00; 16.90]
Random effects model 90 14.00 [1.64; 26.37]
Heterogeneity: I^2 = 68%, τ^2 = 0.0054, χ_1^2 = 3.13 (p = 0.08)
0 5 10 15 20 25 30
Prevalence (%)

J

Study ID	Cases	Total	Prevalence	95% C.I.
**Jiangsu**				
Huang 2020a	12	221	5.43	[2.44; 8.42]

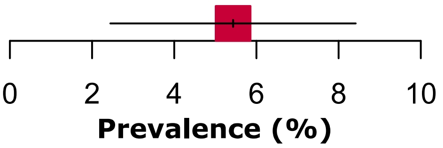

K

Study ID	Cases	Total	Prevalence	95% C.I.
**Sichuan**				
Fu 2020	4	52	7.69	[0.45; 14.93]

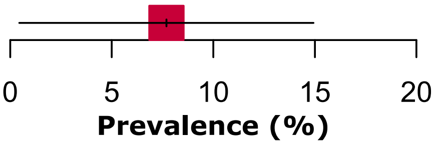

L

Study ID	Cases	Total	Prevalence	95% C.I.
**Hebei**				
Chen 2020d	3	37	8.11	[0; 16.9]

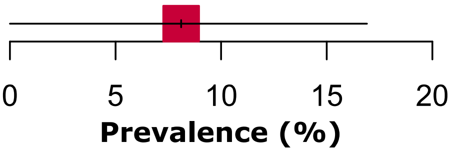

M

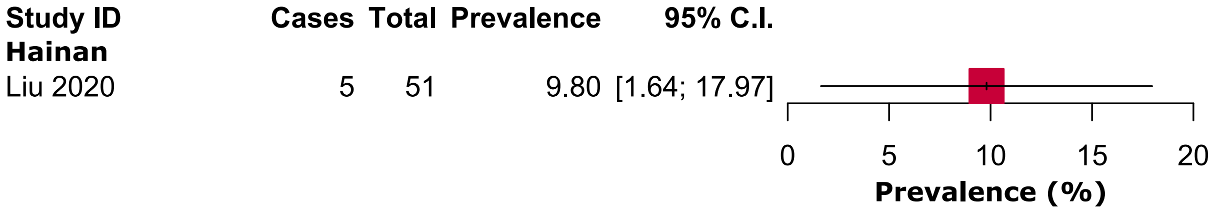

Supplementary Figure 2. Prevalence of headache in COVID-19 patients from (A) Hubei, (B) Shanghai, (C) Zhejiang, (D) Beijing, (E) Chongqing, (F) Guangdong, (G) Anhui, (H) Hunan, (I) Shandong, (J) Jiangsu, (K) Sichuan, (L) Hebei, and (M) Hainan.
