# Supplementary material for: Prevalence of Headache in Patients With Coronavirus Disease 2019 (COVID-19): A Systematic Review and Meta-Analysis of 14,275 Patients
Source: Front Neurol. 2020 Nov 27;11:562634. doi: 10.3389/fneur.2020.562634 (PMC7728918; doi:10.3389/fneur.2020.562634)
Supplement: Supplementary file 10 [file Image_3.PDF]

A

Study ID	Cases	Total	Prevalence	95% C.I.
**Severe or critical**				
Bhatraju 2020	2	24	8.33	[0.00; 19.39]
Chen 2020	1	10	10.00	[0.00; 28.59]
Chen 2020d	3	37	8.11	[0.00; 16.90]
Du 2020	16	100	16.00	[8.81; 23.19]
Feng 2020	1	15	6.67	[0.00; 19.29]
Hu 2020	0	146	0.00	[0.00; 0.94]
Huang 2020a	0	25	0.00	[0.00; 5.28]
Li 2020a	3	47	6.38	[0.00; 13.37]
Li 2020b	3	25	12.00	[0.00; 24.74]
Liu 2020a	0	7	0.00	[0.00; 16.77]
Liu 2020d	2	13	15.38	[0.00; 35.00]
Lu 2020a	1	22	4.55	[0.00; 13.25]
Qin 2020	39	286	13.64	[9.66; 17.61]
Wan 2020	11	40	27.50	[13.66; 41.34]
Wang 2020a	0	11	0.00	[0.00; 11.31]
Xu 2020a	5	25	20.00	[4.32; 35.68]
Yang 2020	3	52	5.77	[0.00; 12.11]
Zhang 2020	0	35	0.00	[0.00; 3.82]
Zhang 2020a	4	55	7.27	[0.41; 14.14]
**Random effects model**		**975**	**7.40**	**[3.93; 10.87]**

Heterogeneity: I^2 = 82%, τ^2 = 0.0036, χ^2_{18} = 97.49 (p < 0.01)

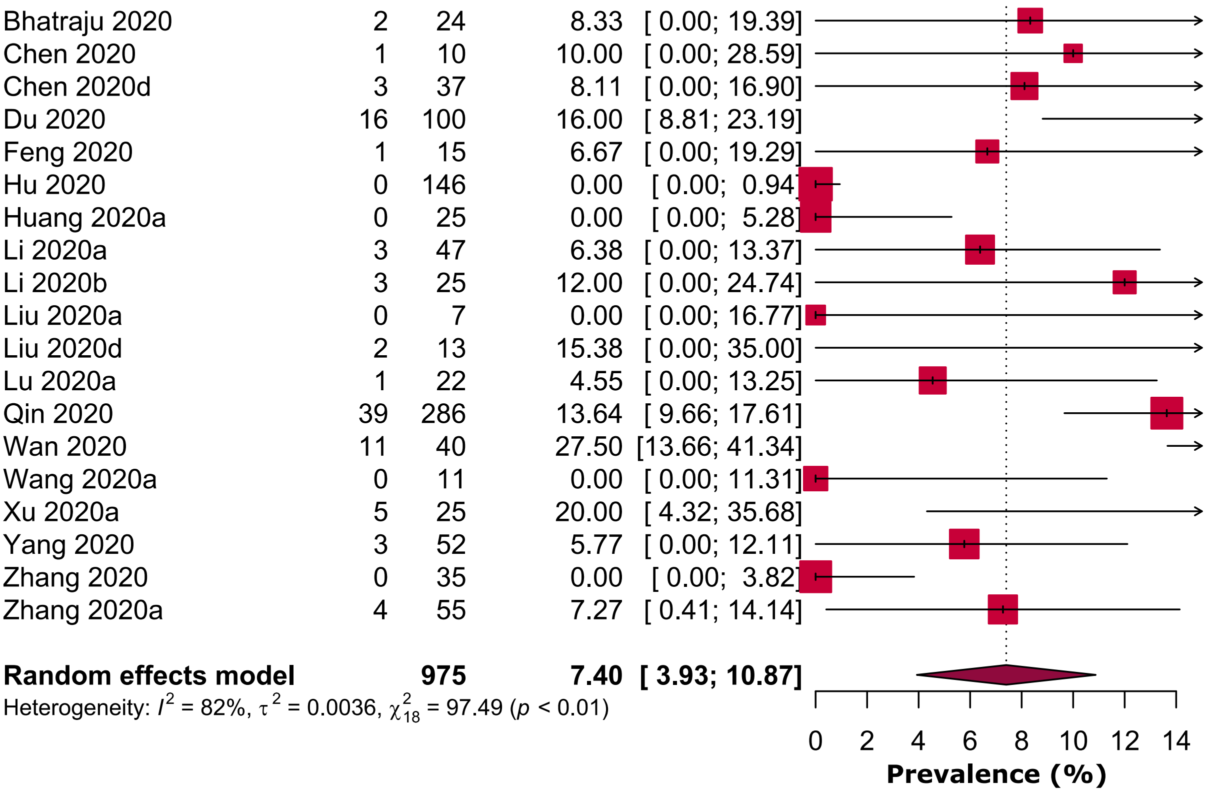

B

Study ID	Cases	Total	Prevalence	95% C.I.
**Non-severe**				
Chen 2020	1	10	10.00	[0.00; 28.59]
Du 2020	3	79	3.80	[0.00; 8.01]
Feng 2020	4	126	3.17	[0.11; 6.24]
Hu 2020	3	151	1.99	[0.00; 4.21]
Huang 2020a	12	196	6.12	[2.77; 9.48]
Li 2020b	6	58	10.34	[2.51; 18.18]
Liu 2020a	5	44	11.36	[1.99; 20.74]
Liu 2020d	6	27	22.22	[6.54; 37.90]
Lu 2020a	25	243	10.29	[6.47; 14.11]
Qin 2020	13	166	7.83	[3.74; 11.92]
Wan 2020	23	95	24.21	[15.60; 32.82]
Wei 2020	5	100	5.00	[0.73; 9.27]
Xu 2020a	18	44	40.91	[26.38; 55.44]
Zhang 2020	2	46	4.35	[0.00; 10.24]
Zhang 2020a	13	166	7.83	[3.74; 11.92]
**Random effects model**		**1551**	**8.63**	**[5.74; 11.51]**

Heterogeneity: $I^2 = 80\%$, $\tau^2 = 0.0021$, $\chi^2_{14} = 69.60$ ($p < 0.01$)

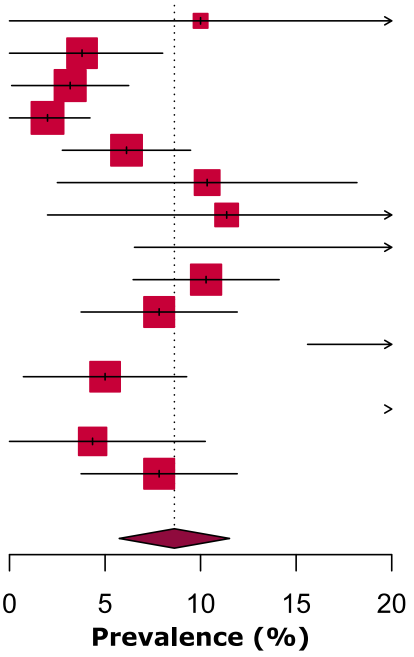

C

Study ID	Cases	Total	Prevalence	95% C.I.
**Survived (recovered or discharged)**				
Chen 2020g	20	161	12.42	[7.33; 17.52]
Deng 2020	7	116	6.03	[1.70; 10.37]
Ding 2020	2	5	40.00	[0.00; 82.94]
Fu 2020	4	52	7.69	[0.45; 14.93]
Han 2020a	2	17	11.76	[0.00; 27.08]
Li 2020	5	96	5.21	[0.76; 9.65]
Li 2020d	6	78	7.69	[1.78; 13.61]
Liu 2020a	5	51	9.80	[1.64; 17.97]
Lu 2020	10	92	10.87	[4.51; 17.23]
Wang 2020d	12	274	4.38	[1.96; 6.80]
Zhang 2020d	18	273	6.59	[3.65; 9.54]
**Random effects model**		**1215**	**7.14**	**[5.30; 8.99]**

Heterogeneity: $I^2 = 29\%$, $\tau^2 = 0.0002$, $\chi^2_{10} = 13.99$ ($p = 0.17$)

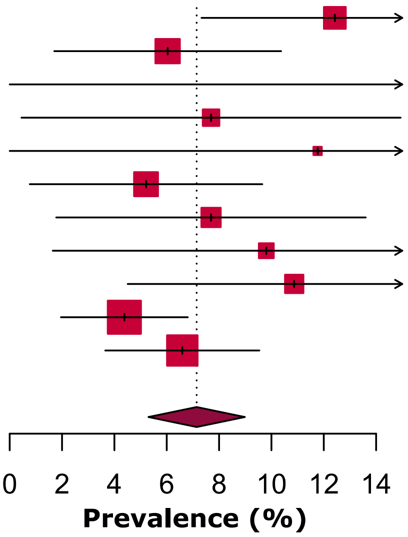

D

Study ID	Cases	Total	Prevalence	95% C.I.
**Non-survived**				
Chen 2020g	11	113	9.73	[4.27; 15.20]
Deng 2020	6	109	5.50	[1.22; 9.79]
Fan 2020	3	101	2.97	[0.00; 6.28]
Li 2020	4	65	6.15	[0.31; 12.00]
Lu 2020	1	31	3.23	[0.00; 9.45]
Wang 2020d	0	65	0.00	[0.00; 2.09]
Zhang 2020d	0	46	0.00	[0.00; 2.93]
**Random effects model**		**530**	**3.31**	**[0.78; 5.83]**

Heterogeneity: $I^2 = 67\%$, $\tau^2 = 0.0007$, $\chi^2_6 = 17.99$ ($p < 0.01$)

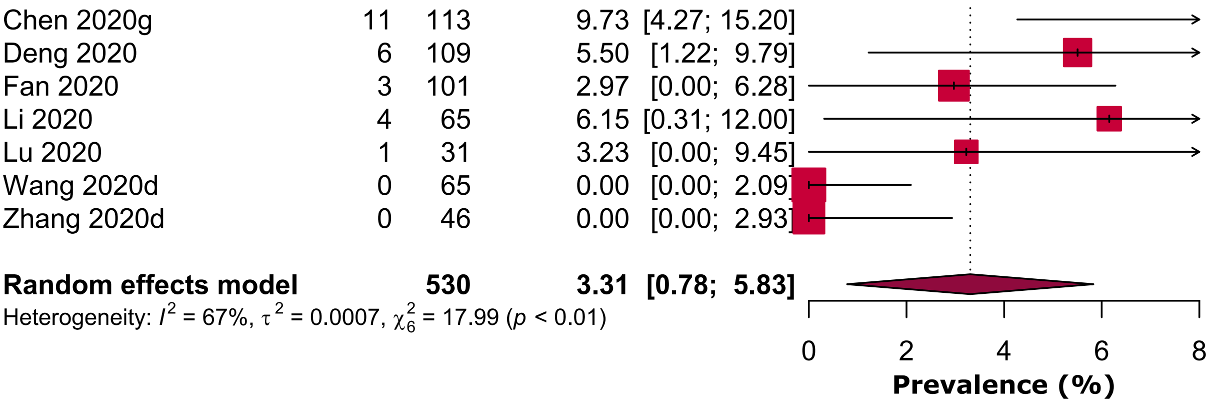

E

Study ID	Cases	Total	Prevalence	95% C.I.
**ICU**				
Cao 2020	0	19	0.00	[0.00; 6.84]
Huang 2020	0	13	0.00	[0.00; 9.72]
Qin 2020a	7	36	19.44	[6.52; 32.37]
Wang 2020	3	36	8.33	[0.00; 17.36]
**Random effects model**		**104**	**5.85**	**[0.00; 13.62]**

Heterogeneity: $I^2 = 64\%$, $\tau^2 = 0.0039$, $\chi^2_3 = 8.31$ ($p = 0.04$)

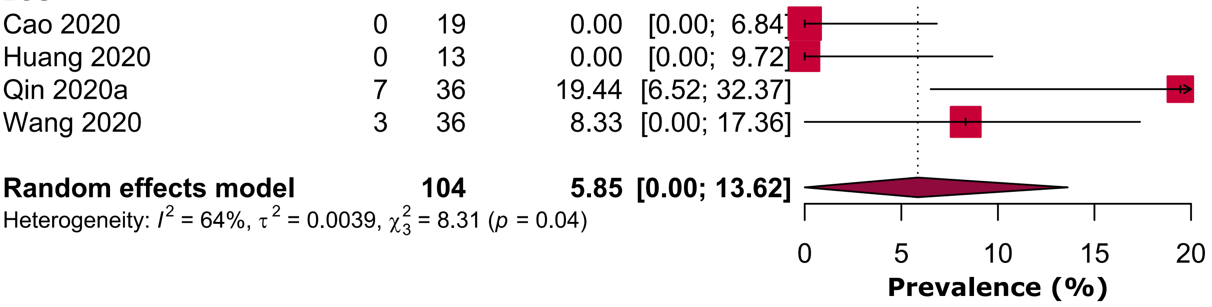

F

Study ID	Cases	Total	Prevalence	95% C.I.
**Non-ICU**				
Cao 2020	24	179	13.41	[8.42; 18.40]
Huang 2020	3	28	10.71	[0.00; 22.17]
Qin 2020a	8	53	15.09	[5.46; 24.73]
Wang 2020	6	102	5.88	[1.32; 10.45]
**Random effects model**		**362**	**10.64**	**[5.81; 15.46]**

Heterogeneity: $I^2 = 50\%$, $\tau^2 = 0.0011$, $\chi^2_3 = 6.00$ ($p = 0.11$)

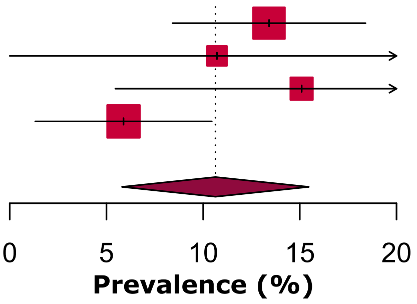

G

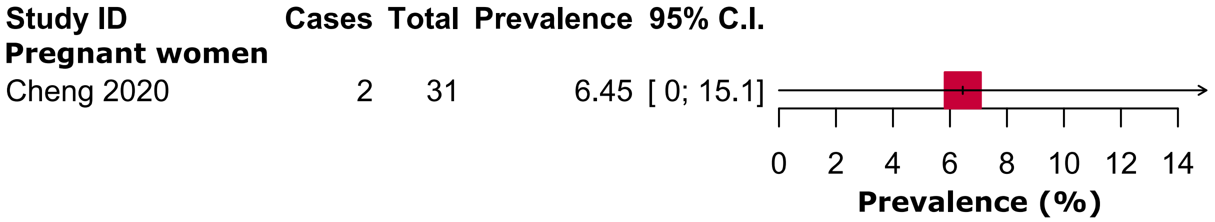

Supplementary Figure 3. Prevalence of headache in (A) severe or critical, (B) non-severe, (C) survived (recovered or discharged), (D) non-survived, (E) ICU, (F) non-ICU and (G) pregnant women with COVID-19.
