# Supplementary material for: Prevalence of Headache in Patients With Coronavirus Disease 2019 (COVID-19): A Systematic Review and Meta-Analysis of 14,275 Patients
Source: Front Neurol. 2020 Nov 27;11:562634. doi: 10.3389/fneur.2020.562634 (PMC7728918; doi:10.3389/fneur.2020.562634)
Supplement: Supplementary file 11 [file Image_4.PDF]

A

Study ID	Cases	Total	Prevalence	95% C.I.
**Excluding small studies**				
Cao 2020	24	198	12.12	[7.58; 16.67]
Chen 2020a	28	249	11.24	[7.32; 15.17]
Chen 2020b	121	534	22.66	[19.11; 26.21]
Chen 2020f	21	123	17.07	[10.42; 23.72]
Chen 2020g	31	274	11.31	[7.56; 15.06]
Cheng 2020	2	111	1.80	[0.00; 4.28]
Deng 2020	13	225	5.78	[2.73; 8.83]
Du 2020	17	179	9.50	[5.20; 13.79]
Fan 2020	3	101	2.97	[0.00; 6.28]
Feng 2020	5	141	3.55	[0.49; 6.60]
Guan 2020	150	1099	13.65	[11.62; 15.68]
Guan 2020a	205	1328	15.44	[13.49; 17.38]
Han 2020	14	108	12.96	[6.63; 19.30]
Hu 2020	3	323	0.93	[0.00; 1.97]
Huang 2020a	12	221	5.43	[2.44; 8.42]
Jin 2020	67	651	10.29	[7.96; 12.63]
Li 2020	9	161	5.59	[2.04; 9.14]
Lian 2020	75	788	9.52	[7.47; 11.57]
Liu 2020b	53	291	18.21	[13.78; 22.65]
Liu 2020f	13	137	9.49	[4.58; 14.40]
Lu 2020	11	123	8.94	[3.90; 13.99]
Lu 2020a	26	265	9.81	[6.23; 13.39]
Mao 2020	23	188	12.23	[7.55; 16.92]
Mo 2020	8	155	5.16	[1.68; 8.64]
Qin 2020	52	452	11.50	[8.56; 14.45]
Shi 2020a	5	101	4.95	[0.72; 9.18]
Shu 2020	52	545	9.54	[7.07; 12.01]
Wan 2020	34	135	25.19	[17.86; 32.51]
Wang 2020	9	138	6.52	[2.40; 10.64]
Wang 2020d	12	339	3.54	[1.57; 5.51]
Wei 2020	5	100	5.00	[0.73; 9.27]
Zhang 2020a	17	221	7.69	[4.18; 11.21]
Zhang 2020b	67	645	10.39	[8.03; 12.74]
Zhang 2020c	60	212	28.30	[22.24; 34.37]
Zhang 2020d	19	319	5.96	[3.36; 8.55]
Zhao 2020b	31	459	6.75	[4.46; 9.05]
Zhou 2020	28	254	11.02	[7.17; 14.88]
**Random effects model**		**11893**	**9.76**	**[7.90; 11.62]**

Heterogeneity: I^2 = 93%, τ^2 = 0.0030, χ^2_{36} = 528.63 (p < 0.01)

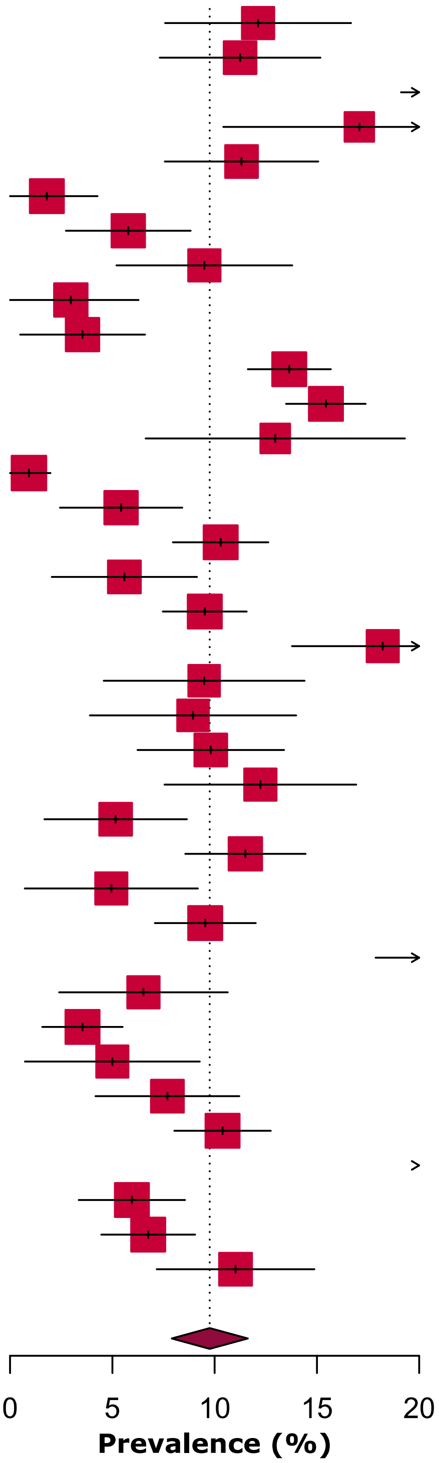

B

Study ID	Cases	Total	Prevalence	95% C.I.
**Excluding low- and moderate-quality studies**				
Cao 2020	24	198	12.12	[7.58; 16.67]
Chen 2020	2	20	10.00	[0.00; 23.15]
Chen 2020a	28	249	11.24	[7.32; 15.17]
Chen 2020b	121	534	22.66	[19.11; 26.21]
Chen 2020e	3	30	10.00	[0.00; 20.74]
Chen 2020f	21	123	17.07	[10.42; 23.72]
Cheng 2020	2	111	1.80	[0.00; 4.28]
Chung 2020	3	21	14.29	[0.00; 29.25]
Ding 2020	2	5	40.00	[0.00; 82.94]
Du 2020	17	179	9.50	[5.20; 13.79]
Du 2020a	11	53	20.75	[9.84; 31.67]
Fan 2020	3	101	2.97	[0.00; 6.28]
Feng 2020	5	141	3.55	[0.49; 6.60]
Guan 2020	150	1099	13.65	[11.62; 15.68]
Guan 2020a	205	1328	15.44	[13.49; 17.38]
Han 2020a	2	17	11.76	[0.00; 27.08]
He 2020	1	31	3.23	[0.00; 9.45]
Hu 2020	3	323	0.93	[0.00; 1.97]
Huang 2020	3	38	7.89	[0.00; 16.47]
Huang 2020a	12	221	5.43	[2.44; 8.42]
Jin 2020	67	651	10.29	[7.96; 12.63]
Li 2020	9	161	5.59	[2.04; 9.14]
Li 2020a	3	47	6.38	[0.00; 13.37]
Li 2020b	9	83	10.84	[4.15; 17.53]
Li 2020c	2	18	11.11	[0.00; 25.63]
Li 2020d	6	78	7.69	[1.78; 13.61]
Lian 2020	75	788	9.52	[7.47; 11.57]
Liang 2020	8	21	38.10	[17.33; 58.87]
Liu 2020b	53	291	18.21	[13.78; 22.65]
Liu 2020c	3	10	30.00	[1.60; 58.40]
Liu 2020d	8	40	20.00	[7.60; 32.40]
Liu 2020e	8	64	12.50	[4.40; 20.60]
Liu 2020f	13	137	9.49	[4.58; 14.40]
Lu 2020	11	123	8.94	[3.90; 13.99]
Lu 2020a	26	265	9.81	[6.23; 13.39]
Mao 2020	23	188	12.23	[7.55; 16.92]
Mo 2020	8	155	5.16	[1.68; 8.64]
Qian 2020	7	91	7.69	[2.22; 13.17]
Shi 2020	5	81	6.17	[0.93; 11.41]
Shi 2020a	5	101	4.95	[0.72; 9.18]
Tian 2020	3	37	8.11	[0.00; 16.90]
Wan 2020	34	135	25.19	[17.86; 32.51]
Wang 2020	9	138	6.52	[2.40; 10.64]
Wang 2020a	0	11	0.00	[0.00; 11.31]
Wang 2020c	10	69	14.49	[6.19; 22.80]
Wang 2020d	12	339	3.54	[1.57; 5.51]
Xu 2020	3	48	6.25	[0.00; 13.10]
Yang 2020a	6	55	10.91	[2.67; 19.15]
Yuanyuan 2020	8	31	25.81	[10.40; 41.21]
Zhang 2020a	17	221	7.69	[4.18; 11.21]
Zhang 2020b	67	645	10.39	[8.03; 12.74]
Zhang 2020c	60	212	28.30	[22.24; 34.37]
Zhang 2020d	19	319	5.96	[3.36; 8.55]
Zhong 2020	11	44	25.00	[12.21; 37.79]
Zhu 2020	0	32	0.00	[0.00; 4.17]
**Random effects model**		**10551**	**10.19**	**[8.39; 11.98]**

Heterogeneity: I^2 = 90%, τ^2 = 0.0034, χ^2_{54} = 564.79 (p < 0.01)

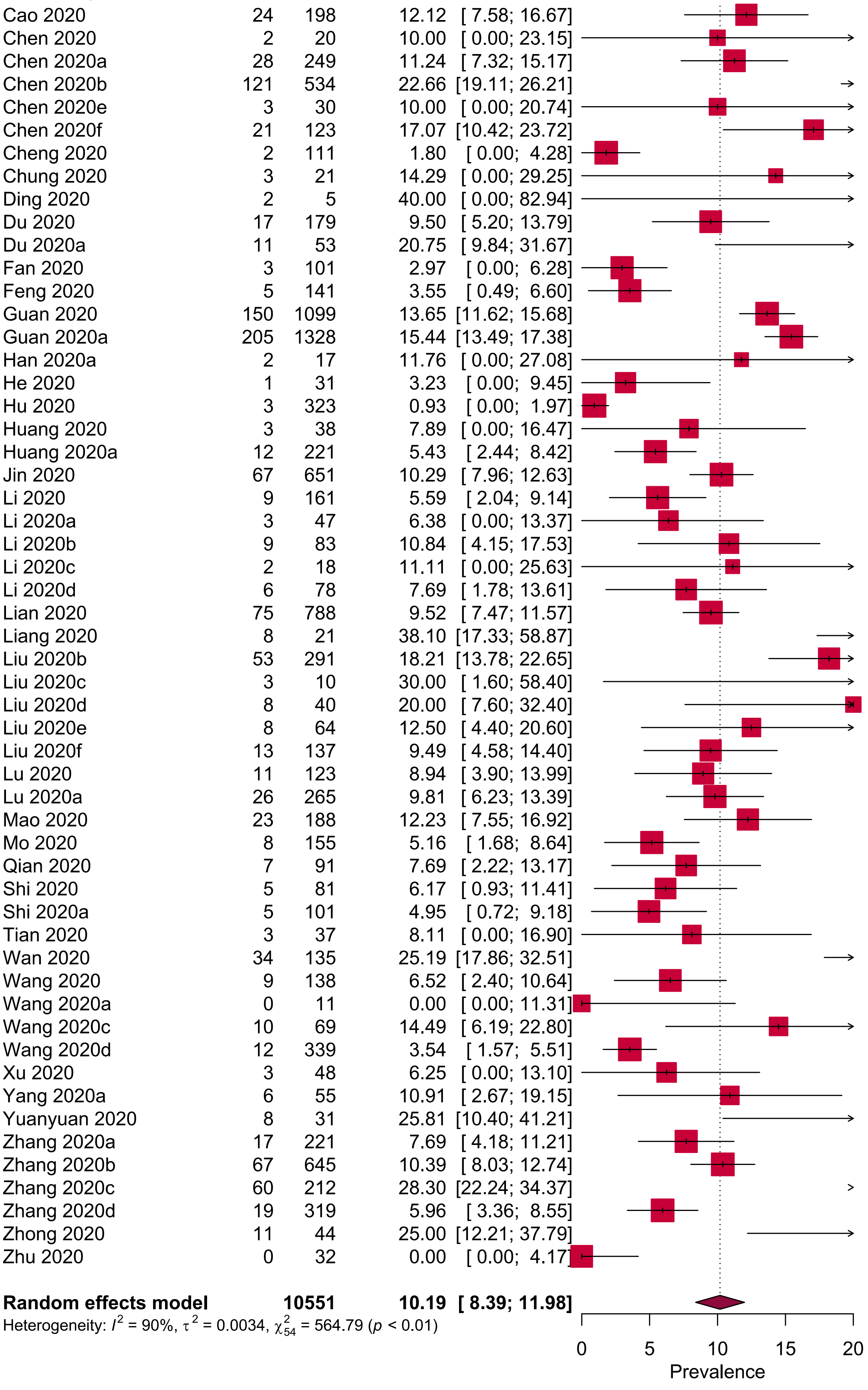

C

Study ID	Cases	Total	Prevalence	95% C.I.
**Excluding studies without reporting COVID-19 confirmation method**				
Bhatraju 2020	2	24	8.33	[0.00; 19.39]
Cao 2020	24	198	12.12	[7.58; 16.67]
Chen 2020	2	20	10.00	[0.00; 23.15]
Chen 2020a	28	249	11.24	[7.32; 15.17]
Chen 2020b	121	534	22.66	[19.11; 26.21]
Chen 2020c	8	99	8.08	[2.71; 13.45]
Chen 2020d	3	37	8.11	[0.00; 16.90]
Chen 2020e	3	30	10.00	[0.00; 20.74]
Chen 2020f	21	123	17.07	[10.42; 23.72]
Chen 2020g	31	274	11.31	[7.56; 15.06]
Cheng 2020	2	111	1.80	[0.00; 4.28]
Deng 2020	13	225	5.78	[2.73; 8.83]
Du 2020	17	179	9.50	[5.20; 13.79]
Du 2020a	11	53	20.75	[9.84; 31.67]
Fan 2020	3	101	2.97	[0.00; 6.28]
Feng 2020	5	141	3.55	[0.49; 6.60]
Fu 2020	4	52	7.69	[0.45; 14.93]
Guan 2020	150	1099	13.65	[11.62; 15.68]
Guan 2020a	205	1328	15.44	[13.49; 17.38]
Han 2020	14	108	12.96	[6.63; 19.30]
Han 2020a	2	17	11.76	[0.00; 27.08]
He 2020	1	31	3.23	[0.00; 9.45]
Hu 2020	3	323	0.93	[0.00; 1.97]
Huang 2020	3	38	7.89	[0.00; 16.47]
Huang 2020a	12	221	5.43	[2.44; 8.42]
Huang 2020b	2	34	5.88	[0.00; 13.79]
Jin 2020	67	651	10.29	[7.96; 12.63]
Li 2020	9	161	5.59	[2.04; 9.14]
Li 2020a	3	47	6.38	[0.00; 13.37]
Li 2020b	9	83	10.84	[4.15; 17.53]
Li 2020c	2	18	11.11	[0.00; 25.63]
Li 2020d	6	78	7.69	[1.78; 13.61]
Lian 2020	75	788	9.52	[7.47; 11.57]
Liang 2020	8	21	38.10	[17.33; 58.87]
Liao 2020	6	30	20.00	[5.69; 34.31]
Liu 2020b	53	291	18.21	[13.78; 22.65]
Liu 2020c	3	10	30.00	[1.60; 58.40]
Liu 2020d	8	40	20.00	[7.60; 32.40]
Liu 2020e	8	64	12.50	[4.40; 20.60]
Liu 2020f	13	137	9.49	[4.58; 14.40]
Lu 2020	11	123	8.94	[3.90; 13.99]
Lu 2020a	26	265	9.81	[6.23; 13.39]
Lu 2020b	7	91	7.69	[2.22; 13.17]
Mao 2020	23	188	12.23	[7.55; 16.92]
Miao 2020	5	62	8.06	[1.29; 14.84]
Mo 2020	8	155	5.16	[1.68; 8.64]
Qian 2020	7	91	7.69	[2.22; 13.17]
Qin 2020	52	452	11.50	[8.56; 14.45]
Qin 2020a	15	89	16.85	[9.08; 24.63]
Shi 2020	5	81	6.17	[0.93; 11.41]
Shu 2020	52	545	9.54	[7.07; 12.01]
Song 2020	8	51	15.69	[5.71; 25.67]
Tian 2020	3	37	8.11	[0.00; 16.90]
Wan 2020	34	135	25.19	[17.86; 32.51]
Wang 2020	9	138	6.52	[2.40; 10.64]
Wang 2020a	0	11	0.00	[0.00; 11.31]
Wang 2020b	4	90	4.44	[0.19; 8.70]
Wang 2020c	10	69	14.49	[6.19; 22.80]
Wang 2020d	12	339	3.54	[1.57; 5.51]
Wei 2020	5	100	5.00	[0.73; 9.27]
Wei 2020a	28	84	33.33	[23.25; 43.41]
Xu 2020	3	48	6.25	[0.00; 13.10]
Xu 2020a	23	69	33.33	[22.21; 44.46]
Yang 2020	3	52	5.77	[0.00; 12.11]
Yang 2020a	6	55	10.91	[2.67; 19.15]
Yao 2020	2	50	4.00	[0.00; 9.43]
Yu 2020	4	76	5.26	[0.24; 10.28]
Yuanyuan 2020	8	31	25.81	[10.40; 41.21]
Zhang 2020	2	81	2.47	[0.00; 5.85]
Zhang 2020a	17	221	7.69	[4.18; 11.21]
Zhang 2020b	67	645	10.39	[8.03; 12.74]
Zhang 2020c	60	212	28.30	[22.24; 34.37]
Zhang 2020d	19	319	5.96	[3.36; 8.55]
Zhao 2020	2	19	10.53	[0.00; 24.33]
Zhao 2020a	5	56	8.93	[1.46; 16.40]
Zhao 2020b	31	459	6.75	[4.46; 9.05]
Zhong 2020	11	44	25.00	[12.21; 37.79]
Zhou 2020	28	254	11.02	[7.17; 14.88]
Zhu 2020	0	32	0.00	[0.00; 4.17]
**Random effects model**		**13987**	**9.98**	**[8.58; 11.37]**

Heterogeneity: $I^2 = 88\%$, $\tau^2 = 0.0029$, $\chi^2_{78} = 653.89$ ($p < 0.01$)

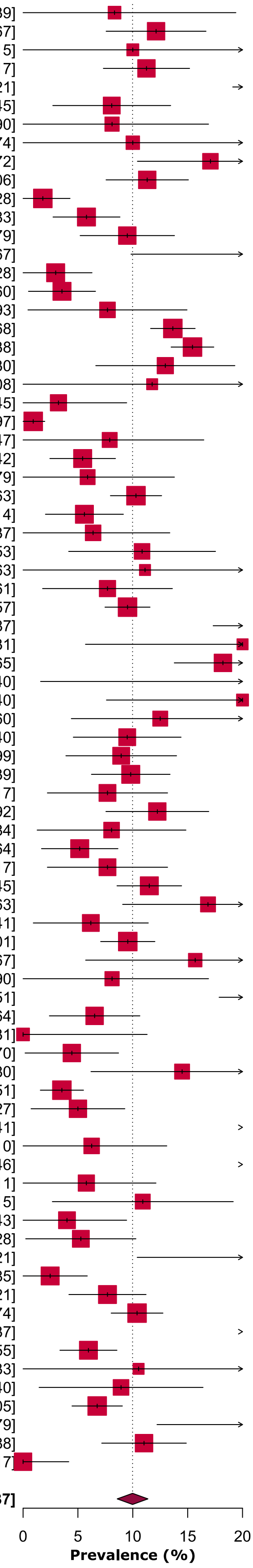

D

Study ID	Cases	Total	Prevalence	95% C.I.
**Excluding non-English studies**				
Bhatraju 2020	2	24	8.33	[0.00; 19.39]
Cao 2020	24	198	12.12	[7.58; 16.67]
Chen 2020	2	20	10.00	[0.00; 23.15]
Chen 2020a	28	249	11.24	[7.32; 15.17]
Chen 2020b	121	534	22.66	[19.11; 26.21]
Chen 2020c	8	99	8.08	[2.71; 13.45]
Chen 2020d	3	37	8.11	[0.00; 16.90]
Chen 2020e	3	30	10.00	[0.00; 20.74]
Chen 2020f	21	123	17.07	[10.42; 23.72]
Chen 2020g	31	274	11.31	[7.56; 15.06]
Cheng 2020	2	111	1.80	[0.00; 4.28]
Chung 2020	3	21	14.29	[0.00; 29.25]
Deng 2020	13	225	5.78	[2.73; 8.83]
Ding 2020	2	5	40.00	[0.00; 82.94]
Du 2020	17	179	9.50	[5.20; 13.79]
Du 2020a	11	53	20.75	[9.84; 31.67]
Fan 2020	3	101	2.97	[0.00; 6.28]
Feng 2020	5	141	3.55	[0.49; 6.60]
Fu 2020	4	52	7.69	[0.45; 14.93]
Guan 2020	150	1099	13.65	[11.62; 15.68]
Guan 2020a	205	1328	15.44	[13.49; 17.38]
Han 2020	14	108	12.96	[6.63; 19.30]
Han 2020a	2	17	11.76	[0.00; 27.08]
He 2020	1	31	3.23	[0.00; 9.45]
Hu 2020	3	323	0.93	[0.00; 1.97]
Huang 2020	3	38	7.89	[0.00; 16.47]
Huang 2020a	12	221	5.43	[2.44; 8.42]
Huang 2020b	2	34	5.88	[0.00; 13.79]
Jin 2020	67	651	10.29	[7.96; 12.63]
Li 2020	9	161	5.59	[2.04; 9.14]
Li 2020a	3	47	6.38	[0.00; 13.37]
Li 2020b	9	83	10.84	[4.15; 17.53]
Li 2020c	2	18	11.11	[0.00; 25.63]
Li 2020d	6	78	7.69	[1.78; 13.61]
Lian 2020	75	788	9.52	[7.47; 11.57]
Liang 2020	8	21	38.10	[17.33; 58.87]
Liao 2020	6	30	20.00	[5.69; 34.31]
Liu 2020	5	51	9.80	[1.64; 17.97]
Liu 2020a	5	51	9.80	[1.64; 17.97]
Liu 2020b	53	291	18.21	[13.78; 22.65]
Liu 2020c	3	10	30.00	[1.60; 58.40]
Liu 2020d	8	40	20.00	[7.60; 32.40]
Liu 2020e	8	64	12.50	[4.40; 20.60]
Liu 2020f	13	137	9.49	[4.58; 14.40]
Lu 2020	11	123	8.94	[3.90; 13.99]
Lu 2020a	26	265	9.81	[6.23; 13.39]
Lu 2020b	7	91	7.69	[2.22; 13.17]
Mao 2020	23	188	12.23	[7.55; 16.92]
Miao 2020	5	62	8.06	[1.29; 14.84]
Mo 2020	8	155	5.16	[1.68; 8.64]
Qian 2020	7	91	7.69	[2.22; 13.17]
Qin 2020	52	452	11.50	[8.56; 14.45]
Qin 2020a	15	89	16.85	[9.08; 24.63]
Shi 2020	5	81	6.17	[0.93; 11.41]
Shi 2020a	5	101	4.95	[0.72; 9.18]
Shu 2020	52	545	9.54	[7.07; 12.01]
Song 2020	8	51	15.69	[5.71; 25.67]
Tian 2020	3	37	8.11	[0.00; 16.90]
Wan 2020	34	135	25.19	[17.86; 32.51]
Wang 2020	9	138	6.52	[2.40; 10.64]
Wang 2020a	0	11	0.00	[0.00; 11.31]
Wang 2020b	4	90	4.44	[0.19; 8.70]
Wang 2020c	10	69	14.49	[6.19; 22.80]
Wang 2020d	12	339	3.54	[1.57; 5.51]
Wei 2020	5	100	5.00	[0.73; 9.27]
Wei 2020a	28	84	33.33	[23.25; 43.41]
Xu 2020	3	48	6.25	[0.00; 13.10]
Xu 2020a	23	69	33.33	[22.21; 44.46]
Yang 2020	3	52	5.77	[0.00; 12.11]
Yang 2020a	6	55	10.91	[2.67; 19.15]
Yao 2020	2	50	4.00	[0.00; 9.43]
Yu 2020	4	76	5.26	[0.24; 10.28]
Zhang 2020	2	81	2.47	[0.00; 5.85]
Zhang 2020a	17	221	7.69	[4.18; 11.21]
Zhang 2020b	67	645	10.39	[8.03; 12.74]
Zhang 2020c	60	212	28.30	[22.24; 34.37]
Zhang 2020d	19	319	5.96	[3.36; 8.55]
Zhao 2020	2	19	10.53	[0.00; 24.33]
Zhao 2020a	5	56	8.93	[1.46; 16.40]
Zhao 2020b	31	459	6.75	[4.46; 9.05]
Zhong 2020	11	44	25.00	[12.21; 37.79]
Zhou 2020	28	254	11.02	[7.17; 14.88]
Zhu 2020	0	32	0.00	[0.00; 4.17]
**Random effects model**		**14185**	**9.86**	**[8.50; 11.21]**

Heterogeneity: $I^2 = 87\%$, $\tau^2 = 0.0028$, $\chi^2_{82} = 653.54$ ($p < 0.01$)

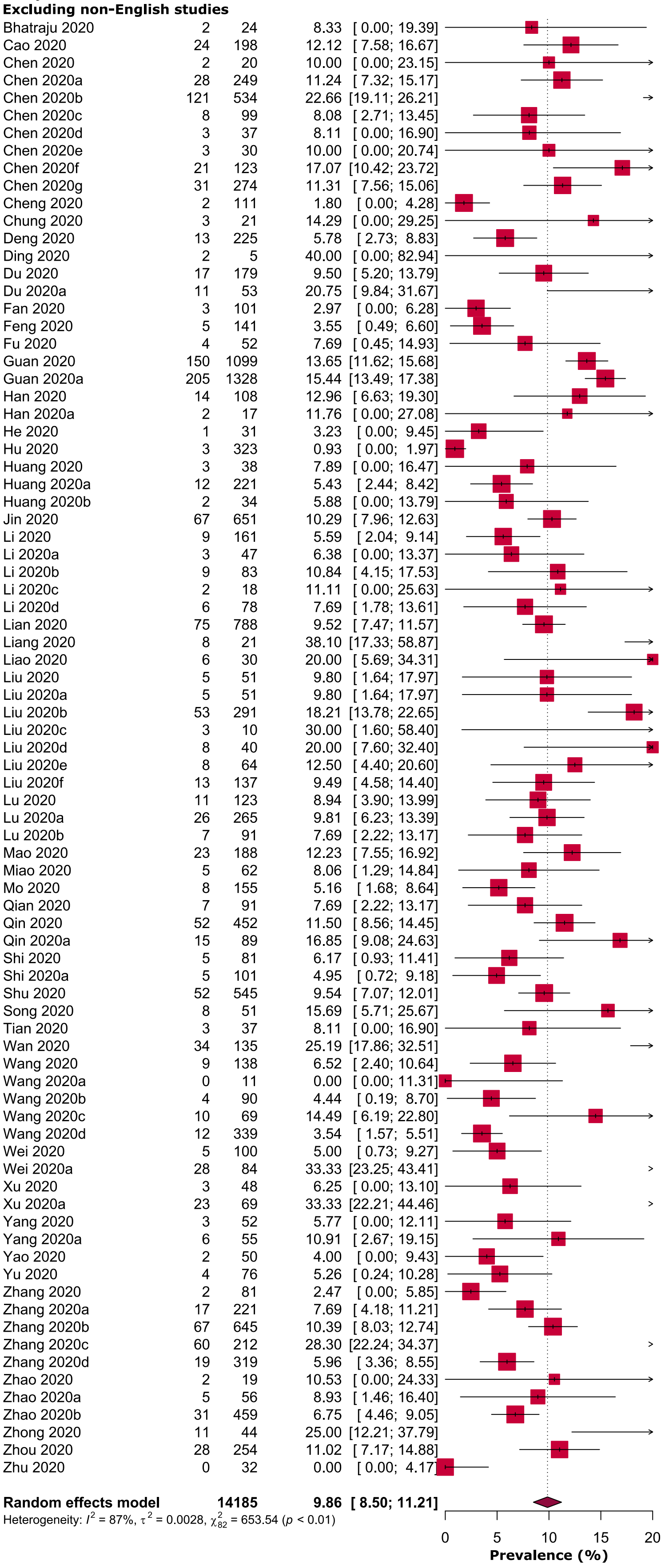

E

Study ID	Cases	Total	Prevalence	95% C.I.
**Excluding outlier studies**				
Bhatraju 2020	2	24	8.33	[0.00; 19.39]
Cao 2020	24	198	12.12	[7.58; 16.67]
Chen 2020	2	20	10.00	[0.00; 23.15]
Chen 2020a	28	249	11.24	[7.32; 15.17]
Chen 2020b	121	534	22.66	[19.11; 26.21]
Chen 2020c	8	99	8.08	[2.71; 13.45]
Chen 2020d	3	37	8.11	[0.00; 16.90]
Chen 2020e	3	30	10.00	[0.00; 20.74]
Chen 2020f	21	123	17.07	[10.42; 23.72]
Chen 2020g	31	274	11.31	[7.56; 15.06]
Cheng 2020	2	111	1.80	[0.00; 4.28]
Chung 2020	3	21	14.29	[0.00; 29.25]
Deng 2020	13	225	5.78	[2.73; 8.83]
Ding 2020	2	5	40.00	[0.00; 82.94]
Du 2020	17	179	9.50	[5.20; 13.79]
Du 2020a	11	53	20.75	[9.84; 31.67]
Fan 2020	3	101	2.97	[0.00; 6.28]
Feng 2020	5	141	3.55	[0.49; 6.60]
Fu 2020	4	52	7.69	[0.45; 14.93]
Guan 2020	150	1099	13.65	[11.62; 15.68]
Guan 2020a	205	1328	15.44	[13.49; 17.38]
Han 2020	14	108	12.96	[6.63; 19.30]
Han 2020a	2	17	11.76	[0.00; 27.08]
He 2020	1	31	3.23	[0.00; 9.45]
Hu 2020	3	323	0.93	[0.00; 1.97]
Huang 2020	3	38	7.89	[0.00; 16.47]
Huang 2020a	12	221	5.43	[2.44; 8.42]
Huang 2020b	2	34	5.88	[0.00; 13.79]
Jin 2020	67	651	10.29	[7.96; 12.63]
Lei 2020	2	29	6.90	[0.00; 16.12]
Li 2020	9	161	5.59	[2.04; 9.14]
Li 2020a	3	47	6.38	[0.00; 13.37]
Li 2020b	9	83	10.84	[4.15; 17.53]
Li 2020c	2	18	11.11	[0.00; 25.63]
Li 2020d	6	78	7.69	[1.78; 13.61]
Lian 2020	75	788	9.52	[7.47; 11.57]
Liao 2020	6	30	20.00	[5.69; 34.31]
Liu 2020	5	51	9.80	[1.64; 17.97]
Liu 2020a	5	51	9.80	[1.64; 17.97]
Liu 2020b	53	291	18.21	[13.78; 22.65]
Liu 2020c	3	10	30.00	[1.60; 58.40]
Liu 2020d	8	40	20.00	[7.60; 32.40]
Liu 2020e	8	64	12.50	[4.40; 20.60]
Liu 2020f	13	137	9.49	[4.58; 14.40]
Lu 2020	11	123	8.94	[3.90; 13.99]
Lu 2020a	26	265	9.81	[6.23; 13.39]
Lu 2020b	7	91	7.69	[2.22; 13.17]
Mao 2020	23	188	12.23	[7.55; 16.92]
Miao 2020	5	62	8.06	[1.29; 14.84]
Mo 2020	8	155	5.16	[1.68; 8.64]
Qian 2020	7	91	7.69	[2.22; 13.17]
Qin 2020	52	452	11.50	[8.56; 14.45]
Qin 2020a	15	89	16.85	[9.08; 24.63]
Shi 2020	5	81	6.17	[0.93; 11.41]
Shi 2020a	5	101	4.95	[0.72; 9.18]
Shu 2020	52	545	9.54	[7.07; 12.01]
Song 2020	8	51	15.69	[5.71; 25.67]
Tian 2020	3	37	8.11	[0.00; 16.90]
Wang 2020	9	138	6.52	[2.40; 10.64]
Wang 2020a	0	11	0.00	[0.00; 11.31]
Wang 2020b	4	90	4.44	[0.19; 8.70]
Wang 2020c	10	69	14.49	[6.19; 22.80]
Wang 2020d	12	339	3.54	[1.57; 5.51]
Wei 2020	5	100	5.00	[0.73; 9.27]
Xu 2020	3	48	6.25	[0.00; 13.10]
Yang 2020	3	52	5.77	[0.00; 12.11]
Yang 2020a	6	55	10.91	[2.67; 19.15]
Yao 2020	2	50	4.00	[0.00; 9.43]
Yu 2020	4	76	5.26	[0.24; 10.28]
Zhang 2020	2	81	2.47	[0.00; 5.85]
Zhang 2020a	17	221	7.69	[4.18; 11.21]
Zhang 2020b	67	645	10.39	[8.03; 12.74]
Zhang 2020d	19	319	5.96	[3.36; 8.55]
Zhao 2020	2	19	10.53	[0.00; 24.33]
Zhao 2020a	5	56	8.93	[1.46; 16.40]
Zhao 2020b	31	459	6.75	[4.46; 9.05]
Zhong 2020	11	44	25.00	[12.21; 37.79]
Zhou 2020	28	254	11.02	[7.17; 14.88]
Zhu 2020	0	32	0.00	[0.00; 4.17]
**Random effects model**		**13693**	**8.84**	**[7.57; 10.10]**

Heterogeneity: I^2 = 85%, τ^2 = 0.0023, χ^2_{78} = 531.13 (p < 0.01)

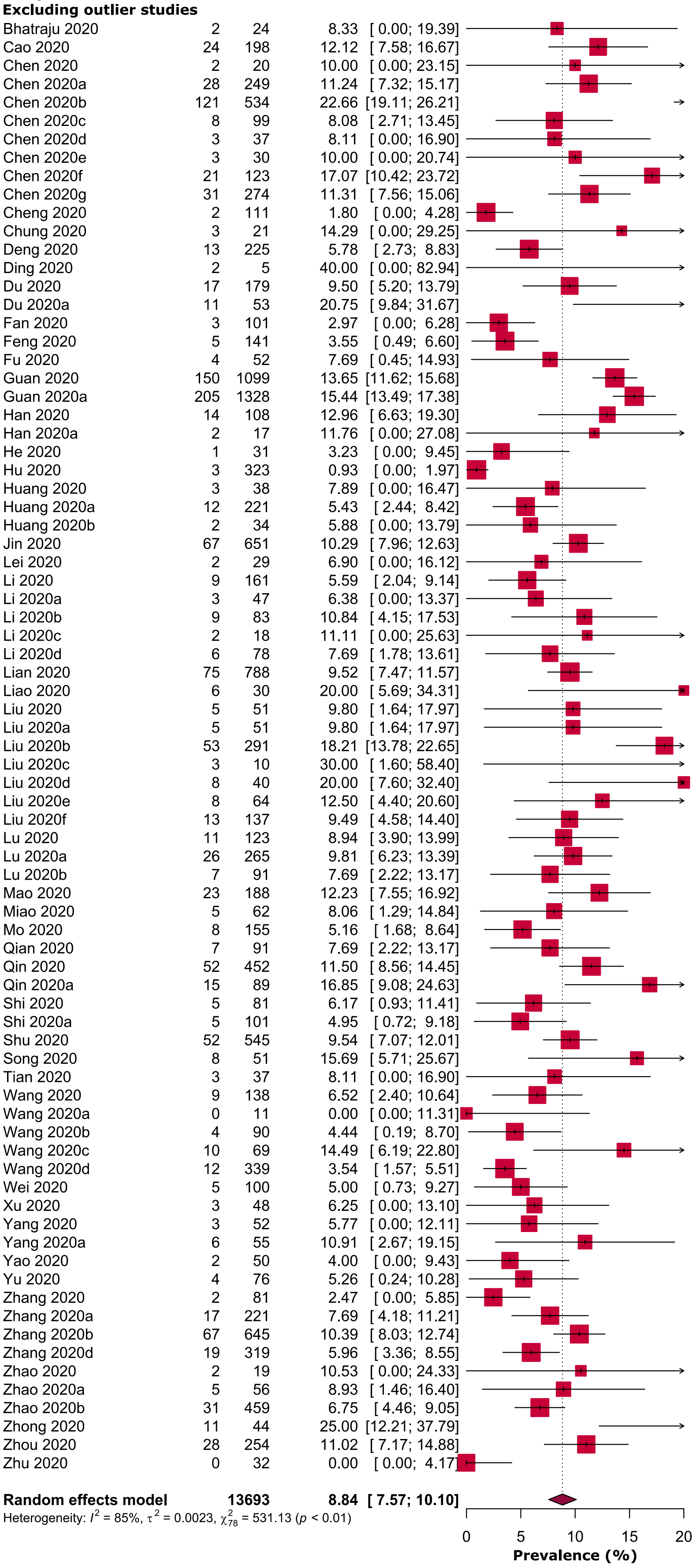

F

Study ID	Cases	Total	Prevalence	95% C.I.
**Considering only cross-sectional studies**				
Bhatraju 2020	2	24	8.33	[0.00; 19.39]
Cao 2020	24	198	12.12	[7.58; 16.67]
Chen 2020	2	20	10.00	[0.00; 23.15]
Chen 2020a	28	249	11.24	[7.32; 15.17]
Chen 2020b	121	534	22.66	[19.11; 26.21]
Chen 2020c	8	99	8.08	[2.71; 13.45]
Chen 2020d	3	37	8.11	[0.00; 16.90]
Chen 2020e	3	30	10.00	[0.00; 20.74]
Chen 2020f	21	123	17.07	[10.42; 23.72]
Chen 2020g	31	274	11.31	[7.56; 15.06]
Deng 2020	13	225	5.78	[2.73; 8.83]
Du 2020	17	179	9.50	[5.20; 13.79]
Du 2020a	11	53	20.75	[9.84; 31.67]
Fan 2020	3	101	2.97	[0.00; 6.28]
Feng 2020	5	141	3.55	[0.49; 6.60]
Fu 2020	4	52	7.69	[0.45; 14.93]
Guan 2020	150	1099	13.65	[11.62; 15.68]
Han 2020	14	108	12.96	[6.63; 19.30]
Hu 2020	3	323	0.93	[0.00; 1.97]
Huang 2020	3	38	7.89	[0.00; 16.47]
Huang 2020a	12	221	5.43	[2.44; 8.42]
Huang 2020b	2	34	5.88	[0.00; 13.79]
Jin 2020	67	651	10.29	[7.96; 12.63]
Lei 2020	2	29	6.90	[0.00; 16.12]
Li 2020	9	161	5.59	[2.04; 9.14]
Li 2020a	3	47	6.38	[0.00; 13.37]
Li 2020b	9	83	10.84	[4.15; 17.53]
Li 2020c	2	18	11.11	[0.00; 25.63]
Li 2020d	6	78	7.69	[1.78; 13.61]
Lian 2020	75	788	9.52	[7.47; 11.57]
Liang 2020	8	21	38.10	[17.33; 58.87]
Liao 2020	6	30	20.00	[5.69; 34.31]
Liu 2020a	5	51	9.80	[1.64; 17.97]
Liu 2020b	53	291	18.21	[13.78; 22.65]
Liu 2020d	8	40	20.00	[7.60; 32.40]
Liu 2020e	8	64	12.50	[4.40; 20.60]
Liu 2020f	13	137	9.49	[4.58; 14.40]
Lu 2020	11	123	8.94	[3.90; 13.99]
Lu 2020b	7	91	7.69	[2.22; 13.17]
Miao 2020	5	62	8.06	[1.29; 14.84]
Min 2020	16	30	53.33	[35.48; 71.19]
Mo 2020	8	155	5.16	[1.68; 8.64]
Qian 2020	7	91	7.69	[2.22; 13.17]
Qin 2020	52	452	11.50	[8.56; 14.45]
Qin 2020a	15	89	16.85	[9.08; 24.63]
Shi 2020	5	81	6.17	[0.93; 11.41]
Shi 2020a	5	101	4.95	[0.72; 9.18]
Shu 2020	52	545	9.54	[7.07; 12.01]
Song 2020	8	51	15.69	[5.71; 25.67]
Tian 2020	3	37	8.11	[0.00; 16.90]
Wan 2020	34	135	25.19	[17.86; 32.51]
Wang 2020	9	138	6.52	[2.40; 10.64]
Wang 2020b	4	90	4.44	[0.19; 8.70]
Wang 2020c	10	69	14.49	[6.19; 22.80]
Wang 2020d	12	339	3.54	[1.57; 5.51]
Wei 2020	5	100	5.00	[0.73; 9.27]
Wei 2020a	28	84	33.33	[23.25; 43.41]
Xu 2020	3	48	6.25	[0.00; 13.10]
Xu 2020a	23	69	33.33	[22.21; 44.46]
Yang 2020	3	52	5.77	[0.00; 12.11]
Yang 2020a	6	55	10.91	[2.67; 19.15]
Yao 2020	2	50	4.00	[0.00; 9.43]
Yu 2020	4	76	5.26	[0.24; 10.28]
Yuanyuan 2020	8	31	25.81	[10.40; 41.21]
Zhang 2020	2	81	2.47	[0.00; 5.85]
Zhang 2020a	17	221	7.69	[4.18; 11.21]
Zhang 2020b	67	645	10.39	[8.03; 12.74]
Zhang 2020c	60	212	28.30	[22.24; 34.37]
Zhang 2020d	19	319	5.96	[3.36; 8.55]
Zhao 2020	2	19	10.53	[0.00; 24.33]
Zhao 2020a	5	56	8.93	[1.46; 16.40]
Zhao 2020b	31	459	6.75	[4.46; 9.05]
Zhou 2020	28	254	11.02	[7.17; 14.88]
Zhu 2020	0	32	0.00	[0.00; 4.17]
**Random effects model**		**12193**	**10.08**	**[8.65; 11.50]**

Heterogeneity: $I^2 = 87\%$, $\tau^2 = 0.0028$, $\chi^2_{73} = 577.06$ ($p < 0.01$)

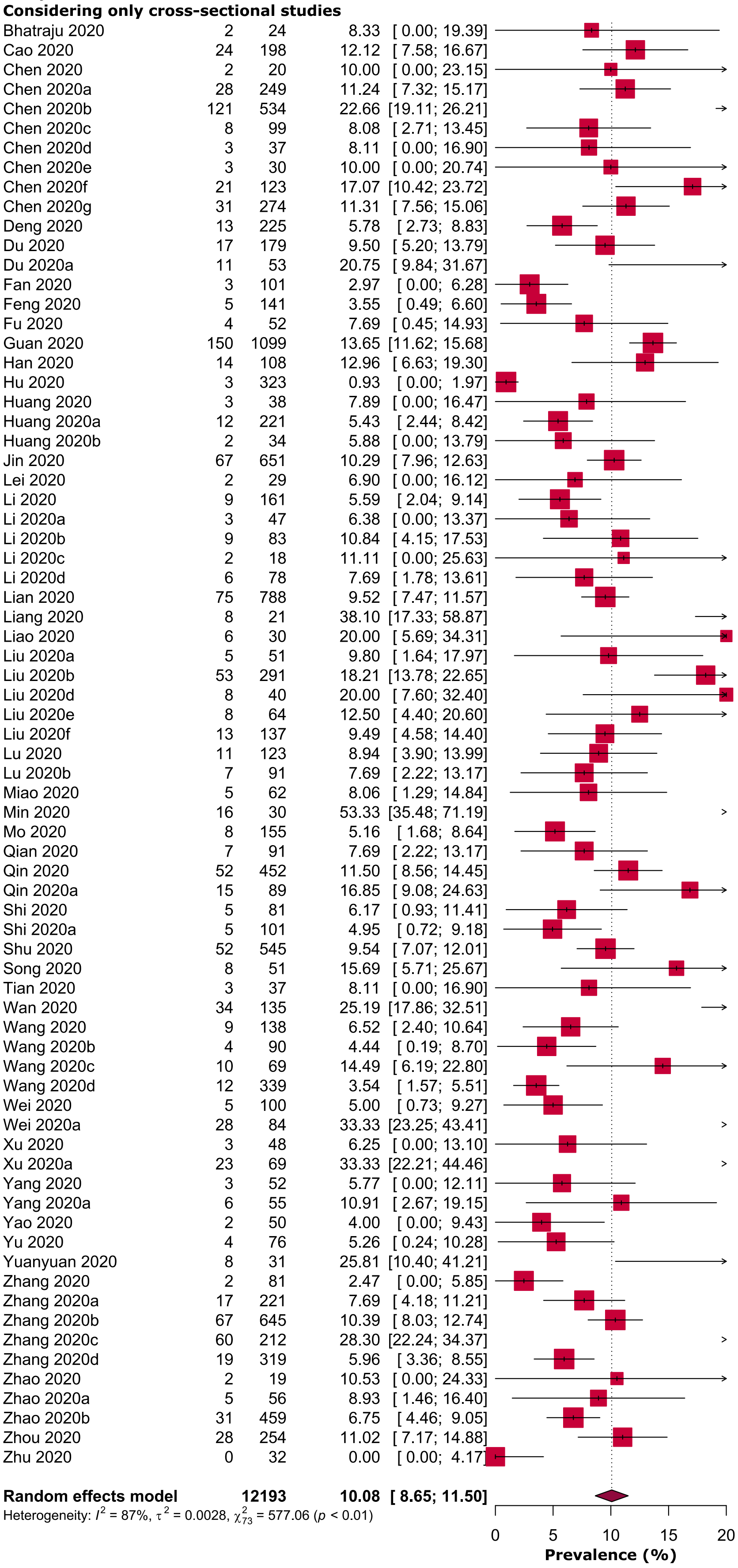

Supplementary Figure 4. Sensitivity analyses: Prevalence of headache in COVID-19 patients (A) excluding small studies (n<100), (B) excluding low- and moderate-quality studies, (C) excluding studies without RT-PCR confirmation or method not reported, (D) excluding non-English studies, (E) excluding outlier studies, and (F) considering only cross-sectional studies.
